# Unveiling the Cold Acclimation of Alfalfa: Insights into Its Starch-Soluble Sugar Dynamic Transformation

**DOI:** 10.3390/plants14091313

**Published:** 2025-04-26

**Authors:** Lin Zhu, Zhiyong Li, Xiaoqing Zhang, Guomei Yin, Siqi Liu, Jinmei Zhao, Ying Yun, Maowei Guo, Jiaqi Zhang

**Affiliations:** 1 Institute of Grassland Research, Chinese Academy of Agricultural Sciences, Hohhot 010011, China; zhuhl1413@126.com (L.Z.); zhiyongli1216@126.com (Z.L.); guomaowei@126.com (M.G.);; 2 Key Laboratory of Forage Resources and Utilization, Ministry of Agriculture and Rural Affairs, Hohhot 010010, China; 3 Institute of Grassland Research, Inner Mongolia Academy of Agricultural and Animal Husbandry Sciences, Hohhot 010031, China

**Keywords:** alfalfa, fall dormancy, field cold acclimation, saccharide transformation, transcriptome

## Abstract

Alfalfa (*Medicago sativa*) is a globally distributed economic legume crop used for forage and ecological restoration. We aimed to explore the mechanisms underlying the cold acclimation observed in this species. Our results for fall plant growth showed that non-dormant alfalfa (SD) maintained a vigorous growth rate compared to that of fall-dormant alfalfa (ZD); however, the winter survival rate of ZD was higher than that of SD. Among the ZD samples, the starch content first accumulated and then decreased; the sucrose content was consumed first along with simultaneous raffinose accumulation, which was followed by sucrose content accumulation, with consistent changes in the corresponding related synthase and hydrolase activity. SD exhibited the opposite trend. The transcriptome data showed that most of the differentially expressed genes were involved in carbon metabolism (ko01200), amino acid biosynthesis (ko01230), and starch and sucrose metabolism (ko00500). Our data clearly show that alfalfa’s cold acclimation mechanism is a complex process, with the establishment of stable carbon homeostasis; sucrose is first converted into starch and raffinose, and then, starch is converted into sucrose, which enables alfalfa’s cold resistance. The process is accompanied by CBF/DREB1A TF regulation. This study provides important insights into the cold acclimation mechanisms of alfalfa.

## 1. Introduction

Alfalfa (*Medicago sativa*) is an economic legume crop grown globally. It is famous for its abundant nutrients and protein is often used as forage for animals that provide meat and milk; it also has excellent nitrogen fixation capabilities for use in ecological restoration, and is a key resource under consideration for future biofuel feedstock applications [1,2,3]. In high-latitude and high-altitude regions, including northern China, cold stress and freezing damage are major limiting factors for alfalfa growth and production [4,5]. Severe cold, particularly in freezing conditions, leads to extracellular ice formation, resulting in plasma membrane damage, osmotic imbalance, and cellular dehydration, ultimately leading to challenges in overwintering and plant death [6,7].

Local northern plants generally adopt several strategies to survive winter: either their roots grow deeply in the soil, or they acquire cold tolerance and survive winter through exposure to low-temperature but non-freezing habitats, which is known as cold acclimation [8,9,10]. Cold acclimation is a critical adaptive strategy involving physiological and metabolic remodeling [11,12], such as cellular constituent modifications through adaptation to seasonal and photoperiod changes to synthesize carbohydrates or other metabolites [9,10]. Recent research has suggested that nonstructural carbohydrates (NSCs) serve a dual role in plants’ cold acclimation. On the one hand, NSCs act as energy sources for maintaining the cellular energy balance and plants’ growth [13]. On the other hand, as osmoregulators and signal molecules for plants’ response to abiotic stress, such as sucrose and raffinose, which are synthesized by sucrose synthase and raffinose synthase, respectively, they facilitate membrane vitrification after water removal and quench reactive oxygen species efficiently, reducing osmotic dehydration and enhancing plasma membrane stability, ultimately preventing cold dehydration and damage [14,15,16]. NSC synthesis has been clearly linked to improved plant cold tolerance.

To date, a number of studies have linked the accumulation of specific sugars during cold stress to improved cold tolerance in various plant species; however, few studies have investigated the interconversion of NSCs for the precise regulation of carbon allocation in alfalfa. In this study, we set out to better understand the cold acclimation mechanisms that enable the higher overwintering survival rate in alfalfa under changes in sunshine duration and intensity variations to freezing temperatures, and we also clarify the changes and transformation of nonstructural carbohydrates.

## 2. Results

### 2.1. Comparison of Plant Growth Differences Between Two Alfalfa Cultivars

**Figure 1:**
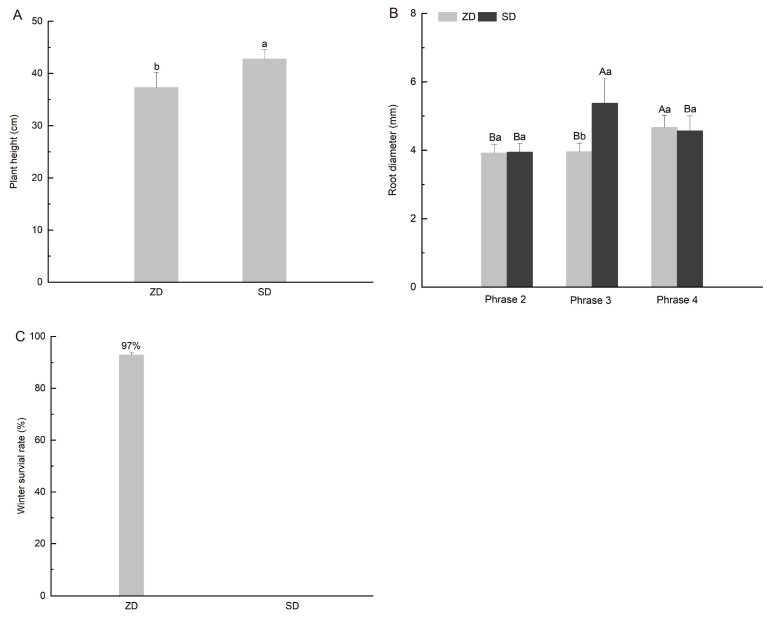
Comparison of plant growth and winter survival rate between two cultivars. Note: (**A**) Plant height difference between two alfalfa cultivars in phase 2 (short daylight condition); different lowercase letters indicate significant differences between the two alfalfa cultivars; (**B**) root diameter difference between the two alfalfa cultivars in phases 2 to 4, different capital letters indicate differences in three phases, and different lowercase letters indicate differences between two cultivars; (**C**) winter survival rate of two alfalfa cultivars observed the year following the commencement of the experiment.

*Medicago sativa* L. ‘Zhaodong’ (ZD) is a native fall-dormant cultivar with good cold tolerance, and *Medicago sativa* L. ‘Sardi No. 10’ (SD) is a non-fall-dormant cultivar adapted to a warm climate. These two alfalfa cultivars with different fall dormancy ratings were used for understanding alfalfa’s growth characteristics and their capacity for environmental adaptation. We assessed the plant height under short daylight conditions (phase 2) and the root diameter changes under conditions of short daylight, temperature cooling down and temperatures below freezing (phases 2 to 4), and the overwintering rates were measured the following year. The most important differences between ZD and SD were that ZD had a shorter plant height (Figure 1A), a thicker root diameter in phase 4 (Figure 1B), and a higher overwintering rate (Figure 1C). The results suggest that minimal growth occurred in ZD entering the cold acclimation stage, in contrast to the continued growth in the SD cultivar. The cold tolerance of ZD was better than that of SD.

### 2.2. Comparison of Dry Matter and Starch Accumulation Between Two Alfalfa Cultivars

**Figure 2:**
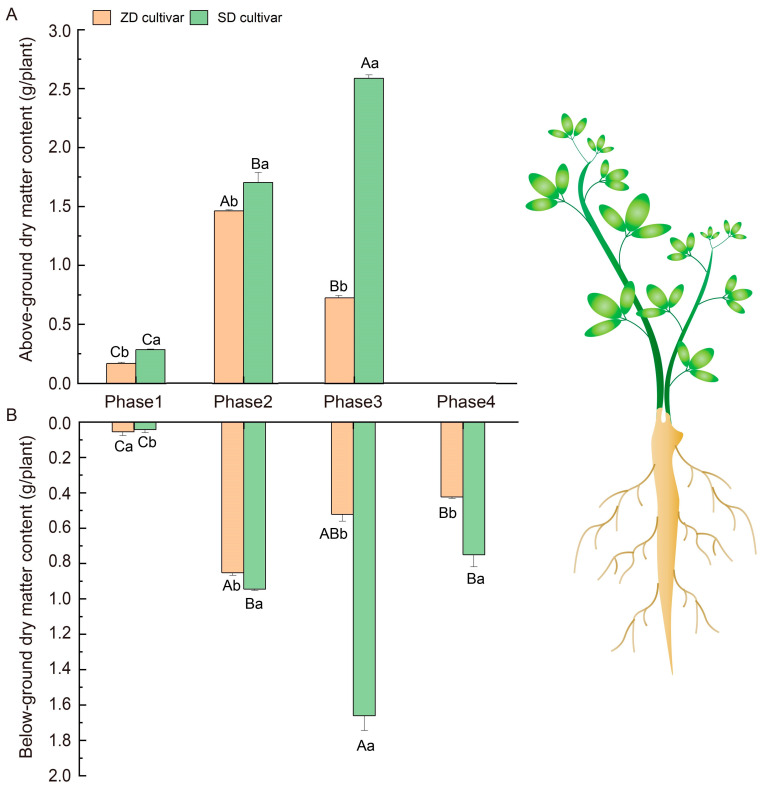
Comparison of above-ground and below-ground dry matter between the two cultivars. Note: (**A**) Plant dry matter of above-ground comparing between two alfalfa cultivars; (**B**) plant dry matter of below-ground comparing between two alfalfa cultivars, different capital letters indicate differences in three phases, and different lowercase letters indicate differences between the two cultivars.

The alfalfa cultivars with different fall dormancy levels exhibited distinct growth characteristics. Further analysis revealed that the ZD exhibited a lower aboveground and belowground dry matter content (Figure 2).

**Figure 3:**
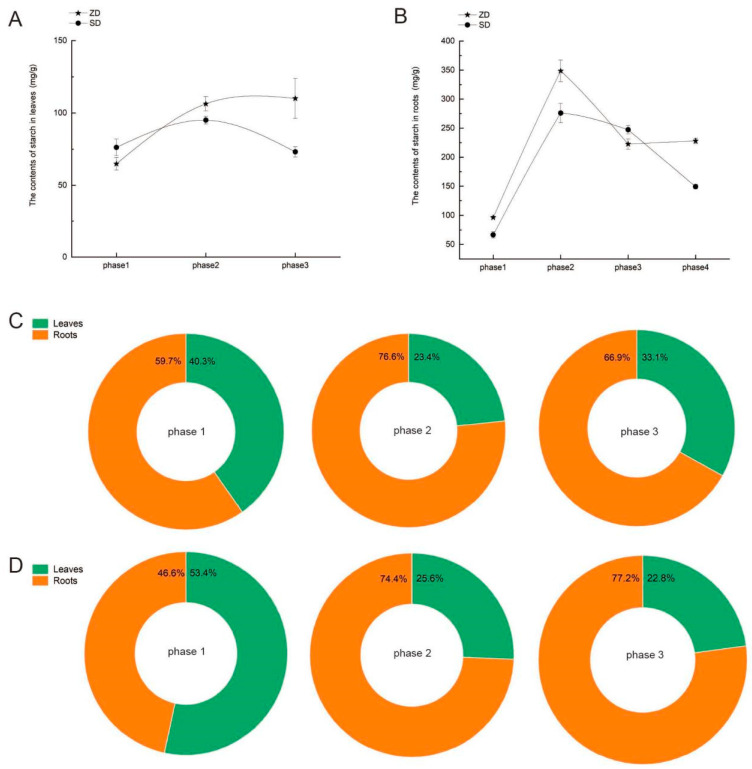
Difference in above-ground and below-ground dry matter accumulation between two alfalfa cultivars in four phases. Note: Figures (**A**) and (**B**) represent the starch content changes in leaves (left) and roots (right), the pentagram symbol represents the ZD, the circular symbol represents the SD cultivar; figures (**C**) and (**D**) represent the starch distribution of the ZD and SD, respectively, green part indicates the proportion of starch content in leaves, and orange part indicates the proportion of starch content in roots.

From phase 2 to phase 4, the ZD showed rapid starch accumulation in their leaves, while the roots initially exhibited starch accumulation followed by consumption, and a stable starch content was maintained during phases 3 to 4 (Figure 3A,B). Both the leaves and roots of the SD exhibited a pattern of initial starch accumulation followed by continuous starch consumption. The ZD exhibited distinct organ-specific proportion differences, with a higher starch content in the roots than in the leaves in phase 1, which significantly differed from the SD (Figure 3C,D).

### 2.3. Comparison of Soluble Sugar Changes Between Two Alfalfa Cultivars

**Figure 4:**
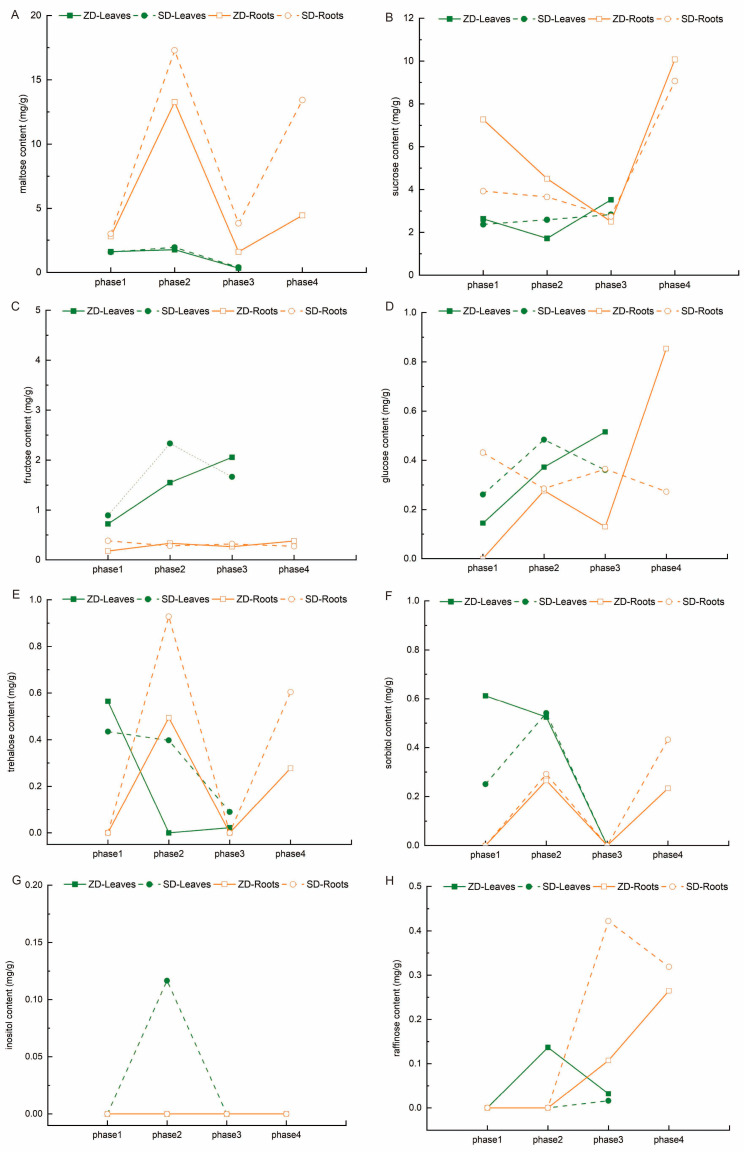
Variation trend of soluble sugars between the two cultivars. Note: Maltose, sucrose, fructose, glucose, sorbitol, inositol, trehalose, and raffinose are shown in (**A**–**H**), respectively. Green: leaf samples; yellow: root samples; solid lines: ZD; dotted lines: SD.

In addition to starch, maltose, sucrose, fructose, glucose, sorbitol, trehalose, inositol, and raffinose are important NSC components. We investigated and compared changes in these eight soluble sugars between the ZD and SD. A comparison of the two cultivars reveals that there were significant differences in the accumulation and proportion of these soluble sugars (Figure 4). From phase 1 to phase 4, there was a slight increase, then a sharp decrease in the maltose content in the alfalfa leaves (Figure 4A and Figure S1), but the maltose content first steeply increased, then steeply decreased, and finally steeply increased again in the roots. It was less accumulated in the ZD than the SD, particularly in phases 3 and 4. This indicates that maltose was mainly consumed in the ZD, used to synthesize starch, or hydrolyzed into glucose.

The sucrose content variation in the ZD is different from that of the SD (Figure 4B and Figure S1). There was a slight decline, then a sharp increase in the ZD’s leaves, whereas there was a slight increase in the SD’s leaves, and the ZD’s leaves accumulated more sucrose content than the SD’s. As for the roots, there was a marked decrease, then a steady increase in both the ZD and SD, in which the ZD accumulated more sucrose content than the SD at phases 1 and 4. These results suggest that sucrose was mainly consumed or hydrolyzed in the ZD during phases 2 and 3, which was the opposite of the phenomenon observed in the SD.

Fructose and glucose are the main products of sucrose hydrolysis and the main substrates for sucrose synthesis, with glucose being the hydrolysate of maltose. The change in the fructose and glucose content can provide a reference for the quantification of maltose and sucrose synthesis or hydrolysis. The fructose content steadily increased in the ZD’s leaves and roots, but gradually dropped in the SD’s leaves and roots, such that the ZD accumulated more fructose content than the SD (Figure 4C and Figure S1). The glucose content gradually rose in the ZD’s leaves; it first markedly increased, then steadily decreased, and finally steeply increased in the ZD’s roots (Figure 4D and Figure S1). The notable differences in the SD included that their glucose content changes were completely opposite to those in the ZD. One phenomenon that emerged from the data was that the fructose and glucose changes shared the same trends. Overall, these results indicate that fructose might be produced by the hydrolysis of sucrose, and glucose might be produced by the hydrolysis of both maltose and sucrose in the ZD. Fructose and glucose might be used to synthesize sucrose, and glucose might be used to synthesize maltose in the SD.

Trehalose is a disaccharide composed of glucose. Sorbitol and inositol are sugar alcohols that can be synthesized from glucose metabolism, and inositol can also be produced from raffinose metabolism. Raffinose is a trisaccharide consisting of galactose and sucrose. In terms of the other obvious differences between the ZD and SD, firstly, the trehalose content was lower in the ZD than that in the SD (Figure 4E and Figure S1). The trend variations in trehalose were similar to those in sucrose in the leaves and glucose in the roots of the ZD, and exhibited an opposite trend to glucose in ZD’s leaves, SD’s leaves, and SD’s roots. Second, the sorbitol content in the ZD was similar to that in the SD (Figure 4F and Figure S1), but with a distinct trend between the ZD’s leaves and SD’s leaves, which involved a gradual decrease in the ZD’s leaves, but an initial slight increase then decrease in the SD’s leaves. The trend in the sorbitol content changed in the roots of the ZD and SD and was found to be consistent with that in the trehalose content. Third, inositol was only accumulated in the SD’s leaves, and raffinose was only accumulated in the ZD’s leaves during phase 2 (Figure 4G,H). Together, these results suggest that trehalose and sorbitol in the ZD were potentially consumed or hydrolyzed into glucose, and sucrose and glucose were used to synthesize raffinose, not inositol.

In addition, the proportion of the maltose content always ranked second in the alfalfa leaves, but it rose from second to first rank in the roots (Figure S1). Whereas the proportion of sucrose content always ranked first in the leaves, it dropped to second rank in the roots. This may be a common phenomenon in alfalfa growth and development.

### 2.4. Comparison of Four Enzyme Activity Changes Between Two Alfalfa Cultivars

**Figure 5:**
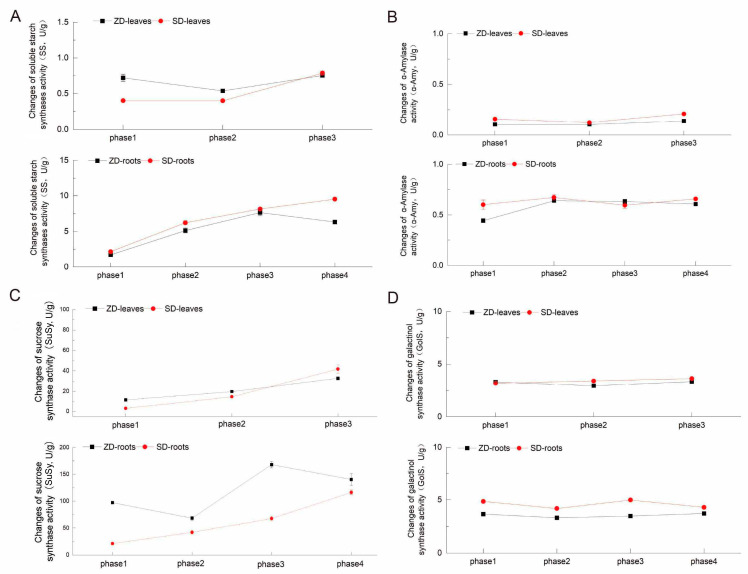
Changes in the activities of four enzymes during the four phases of the two alfalfa cultivars. Note: soluble starch synthase changes, α-Amylase changes, sucrose synthase changes, and galactinol synthase changes are shown in (**A**–**D**), respectively. Black lines with square symbols indicate ZD leaves samples (above) and roots samples (below), and red lines with circular symbols indicate SD leaves samples (above) and roots samples (below).

Soluble starch synthase (SS) is a key enzyme for starch synthesis. The SS activity saw a marked declining trend, then a gradual increase in the leaves of the ZD, but there was first a gradual increase, then a marked decline in the roots (Figure 5A). As for the SD, the SS activity saw a continual increase in both the leaves and roots. The SS activity of the ZD’s leaves was higher than that in the SD’s leaves from phase 1 to phase 2. The SS activity was much higher in the alfalfa roots than in the leaves. The enzyme α-Amylase (α-Amy) is responsible for starch degradation, leading to maltose release. The α-Amy activity saw a continual increase in the leaves of the ZD and SD (Figure 5B), but the α-Amy activity was lower in the ZD than in the SD. As for the roots, the α-Amy activity first increased, then fluctuated slightly in the ZD, whereas it increased slightly then decreased, ultimately showing an increase in the SD; it was only higher in the ZD compared to the SD in phase 3. It was also found that the α-Amy level was higher in the alfalfa roots than in the leaves. But the SS activity was found to be higher than the α-Amy activity in both the alfalfa leaves and roots. Together, these data show that the changing trend in the SS activity and α-Amy activity were consistent with the change trend for starch and maltose.

Sucrose synthase (SuSy) is a key enzyme involved in sucrose metabolism. The SuSy activity gradually increased in the leaves of the ZD and SD; it showed a trend of first receding, then increasing, and ultimately receding again in the ZD’s roots, but it steadily increased in the SD’s roots. The SuSy activity’s changing trend was contrary to the changing trend of fructose and glucose in the ZD, which further indicated that sucrose was hydrolyzed into fructose and glucose in the ZD (Figure 5C).

Galactinol synthase (GoIS) is an enzyme that converts inositol into inositol galactoside; then, inositol galactoside and sucrose can be used for raffinose synthesis. This is a reversible reaction in which inositol galactoside can be converted into inositol by GoIS. The trend in its activity showed a slight decrease, then a slight rise in the ZD’s leaves and roots (Figure 5D). As for the SD, the GoIS activity slightly increased in the leaves, but initially decreased, then increased, and decreased again in the roots. The GoIS activity in the ZD was lower than that in the SD. Its change trend was consistent with the raffinose changes in the alfalfa roots, and was similar to the inositol changes in the SD’s leaves, but contrasted to the raffinose changes in the ZD’s leaves, respectively. These results indicate that galactinol synthase may not be the key enzyme for raffinose synthesis in the ZD, but is still an important enzyme for inositol synthesis in the SD.

### 2.5. Comparison of Transcriptional Changes Between Two Alfalfa Cultivars

Cold acclimatization is a quantitative genetic trait. Freezing tolerance, obtained from cold acclimation, is always accompanied by complex physiological changes that occur after gene expression regulation. Therefore, 14 cDNA libraries were generated from the mRNA of leaves and roots from phases 1 to 4, and these were sequenced using Illumina. In total, over 36,940,636 clean data points were generated, with a GC content > 41.26%, QC score > 91.64%, and >90.93% of the clean data mapped to the reference genome (Table S1). An overview of the assembly results is presented in Table S1. The gene expression analysis conducted using RNA-seq and expectation maximization showed that the gene expression levels varied significantly across different experimental conditions (Figure S2). The correlation analysis between the biological samples indicated a strong replicability, with R2 values close to 1 (Figure S3). 

The subsequent DEG analysis, following the exclusion of the abnormal samples, applied thresholds of FDR ≤ 0.001 and |Log2Fold-Change| ≥ 2; this led to the identification of thousands of DEGs across different growth phases and tissues, and their up-/downregulated changes were then determined. A total of 2646, 7370, and 5039 DEGs were obtained from the three phases of the leaves, and 11,802, 7668, 8318, and 15,929 DEGs were obtained from the four root phases (Table 1). With respect to the leaf transcriptome data, 1018, 4199, and 3629 genes were upregulated in the leaves of phase 1 to phase 3, respectively; 4980, 4260, 5033, and 8731 genes were upregulated in the roots in phase 1 to phase 4, respectively.

### 2.6. Differentially Expressed Gene (DEG) Enrichment Analysis of Two Alfalfa Cultivars

To further investigate the impact of differentially expressed genes in alfalfa during cold acclimation, GO functions were employed to annotate these DEGs, which were classified into three categories and more than 47 GO terms (Figures S4 and S5). Within the biological processes category, the term “metabolic process” (GO:0008152) was enriched for the most DEGs (Tables S2–S8), followed by “cellular process” (GO:0009987) and “single-organism process” (GO:0044699). As for the “metabolic process” term, there were a total of 827 DEGs (317 upregulated and 510 downregulated) in the leaves of phase 1; 2295 DEGs (1319 upregulated and 976 downregulated) in the leaves of phase 2; and 1555 DEGs (1118 upregulated and 437 downregulated) in the leaves of phase 3. There were a total of 3514 DEGs (1567 upregulated and 1947 downregulated) in the roots of phase 1; 2342 DEGs (1210 upregulated and 1132 downregulated) in the roots of phase 2; 2519 DEGs (1541 upregulated and 978 downregulated) in the roots of phase 3 and 4; and 552 DEGs (2284 upregulated and 2268 downregulated) in the roots of phase 4.

Within the cellular component category, the terms “cell” (GO:0005623) and “cell part” (GO:0044464) were enriched for the most DEGs, followed by “organelle” (GO:0043226). For the terms “cell” and “cell part”, there were a total of 443 DEGs (168 upregulated and 275 downregulated) in the leaves of phase 1; 1284 DEGs (739 upregulated and 545 downregulated) in the leaves of phase 2; and 881 DEGs (613 upregulated and 268 downregulated) in the leaves of phase 3. There were a total of 1546 DEGs (710 upregulated and 836 downregulated) in the roots of phase 1; 1219 DEGs (662 upregulated and 557 downregulated) in the roots of phase 2; 1190 DEGs (679 upregulated and 511 downregulated) in the roots of phase 3; and 2502 DEGs (1199 upregulated and 1303 downregulated) in the roots of phase 4.

Within the molecular function category, the term “catalytic activity” (GO:0003824) was enriched for the most DEGs, followed by “binding” (GO:0005488) and “transporter activity” (GO:0005215). For the terms “catalytic activity”, there were a total of 691 DEGs (267 upregulated and 424 downregulated) in the leaves of phase 1; 1984 DEGs (1329 upregulated and 863 downregulated) in the leaves of phase 2; and 1329 DEGs (951 upregulated and 378 downregulated) in the leaves of phase 3. There were a total of 3074 DEGs (1204 upregulated and 1870 downregulated) in the roots of phase 1; 1980 DEGs (1035 upregulated and 945 downregulated) in the roots of phase 2; 2112 DEGs (1286 upregulated and 826 downregulated) in the roots of phase 3; and 4001 DEGs (2046 upregulated and 1955 downregulated) in the roots of phase 4.

**Figure 6:**
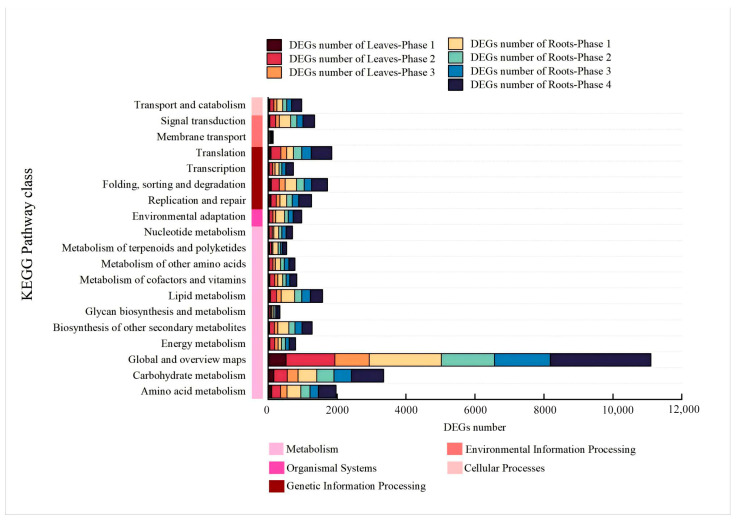
KEGG enrichment analysis of DEGs. Note: Performing KEGG annotation of 34,185 DEGs. There were more than 614 DEGs exposed to 20 terms of 5 categories, including metabolism, organismal systems, environmental information processing, genetic information processing, and cellular processes.

In total, 34,185 DEGs were enriched in five categories, 20 terms, and more than 127 KEGG pathways (Figure 6, Tables S9–S15). Numerous genes were found to be highly concentrated in the subcategories of “carbohydrate metabolism” (for at least 163 DEGs and at most 933 DEGs) and “global and overview maps” (for at least 519 DEGs and at most 2922 DEGs) (Figure 6). As for the subcategory “carbohydrate metabolism”, these DEGs were categorized into 14 KEGG pathways. The KEGG pathways “starch and sucrose metabolism” (ko00500, varied from 17 to 136 DEGs), “amino sugar and nucleotide sugar metabolism” (ko00520, varied from 17 to 140 DEGs), and “glycolysis/gluconeogenesis” (ko00010, varied from 16 to 104 DEGs) were enriched for the majority of the DEGs. As for the subcategory “global and overview maps”, the KEGG pathway “metabolic pathways” (ko01100, varied from 265 to 1450 DEGs), and “biosynthesis of secondary metabolites” (ko01100, varied from 158 to 907 DEGs) were enriched for the majority of the DEGs.

In the pathway ‘’starch and sucrose metabolism’’, most of the DEGs were linked to the synthesis and degradation of starch (*SS* and *BAM*) and sucrose (*SPS* and *SUS*) (Tables S9–S15). The majority of the *SS*s were upregulated in the ZD leaves (especially in phase 1), but were downregulated in their roots (especially in phases 3 and 4) (Figures S6 and S7). Eleven *SS*s were found to be activated in the leaves and under short daylight conditions (phase 2), and sixteen *SS*s were upregulated in the roots at phase 2, whereas they were downregulated after a temperature decrease. A quantity of *BAM*s were upregulated in both the leaves and roots of the ZD, particularly in phase 2 (Figures S8 and S9); 30 *BAM*s in the leaves and 31 *BAM*s in the roots were upregulated in the ZD leaves under short daylight conditions (phase 2). A total of 35 *BAM*s in the leaves and 27 *BAM*s in the roots were upregulated when the temperature cooled (phase 3). Coincidentally, these changes were similar to the trend in starch accumulation in the ZD.

Numerous *SPS*s were upregulated in the ZD leaves during phase 2, while most of them were downregulated in the ZD roots during phase 2 and were upregulated in phases 3 and 4 (Figures S10 and S11). These change trends were similar to those seen in the sucrose synthase activity changes. The majority of the *SUS*s were upregulated in the ZD leaves during phase 1 and phase 3 (Figures S12 and S13), while most of them were upregulated in the ZD roots during phase 1, phase 3, and phase 4; there was a lot of similarity in the changes between the *SUS* expression and sucrose content. There were more upregulated *SUS*s in the leaves than in the roots. Raffinose is a unique soluble sugar only produced in the ZD leaves during phase 2, which is in clear contrast with the SD. The *RFS* gene encodes raffinose synthase and was mostly found to be upregulated in the ZD leaves and roots during phase 2 (Figures S14 and S15), which was consistent with the raffinose changes.

Transcription factors (TFs) often regulate gene expression through a plant’s external stimulation responses, and cold acclimation can induce rapid *CBF/DREB* responses and activation. A total of 142 DEGs associated with *CBF/DREB* were identified (Tables S16–S22), including the following gene family members: 39 *DREB1A*s, 3 *DREB1B*s, 14 *DREB1C*s, 15 *DREB1D*s, 4 *DREB1E*s, 4 *DREB1F*s, 15 *DREB2A*s, 4 *DREB2B*s, 9 *DREB2D*s, 10 *DREB2F*s, 1 *DREB2G*, and 24 *DREB3*s. *DREB1A* seems to play a crucial role in alfalfa cold acclimation. Most of these *DREB1A*s were upregulated in the ZD leaves at phase 2 and roots at phase 1 (Figure S16). This suggests that *DREB1A* plays a crucial role in cold acclimation. Among these *DREB1A*s, four DEGs were significantly activated under short daylight conditions (Figure S17 and S18), including MS.gene30852 and MS.gene49255, which were upregulated in the leaves, and MS.gene34447 and MS.gene49252, which were upregulated in the roots. When the temperature decreased (phase 3), MS.gene34447 was found to be upregulated in the leaves; MS.gene30852 and MS.gene49252 were found to be upregulated in the roots; and MS.gene49255 was found to be upregulated in both the leaves and roots. Cold acclimation under natural conditions induces *CBF/DREB1A* control, which is key for the initiation of survival under seasonally cold temperatures in plants. Primers specific for *SS1*, *BAM3*, *SPS*, *SUS2*, *RFS*, and *DREB1A* were used for RT-qPCR to validate the reliability of the transcriptome data (Figure S19), which were selected from 101 DEGs, including the *BAM*, *SS*, *SUS*, *SPS*, and *RFS* DEGs. This was consistent with the RNA-Seq results, which confirmed the reliability of our RNA-Seq data.

## 3. Discussion

Extreme climates occur frequently on a global scale, and higher temperatures in summer, lower temperatures in winter, and seasonal frost lead to synchronous crop failures globally. Crop production in northern latitudes is easily affected by chilling or freezing temperatures, and many local plant species develop and strengthen their low-temperature tolerance following exposure and adaptation to low (but not freezing) temperatures. The coupling of a high alfalfa yield and strong winter survival capability remains a topic for future research as a strategy for enhancing production.

During cold acclimation, variations in sunshine duration and intensity in early autumn induce a dormant state in plants; subsequently, plants develop cold adaptability from gradual temperature decreases, and, eventually, they are able to survive below 0 °C and re-grow the following year [9,10]. The existing research on alfalfa cold stress is extensive and focuses on simulation experiments, ignoring natural sunlight changes; however, it has been suggested that field sunlight signals are essential for complete cold acclimation [10,17,18,19,20]. To better understand the mechanism of alfalfa’s complete cold acclimation, we present new evidence for the accumulation and transformation of nonstructural carbohydrates, using two alfalfa cultivars with different fall dormancy levels treated under natural seasonal conditions. The fall-dormant cultivar ‘Zhaodong’ (ZD) is a cultivar native to northern China [17]. Contrary to the SD, the ZD was characterized by a lower plant height, lower above- and belowground dry matter values following sunlight signals changing (Figure 1 and Figure 2). However, the ZD root diameter was comparatively larger in the low-temperature phase, which could enable survival in winter (Figure 1 and Figure 2). Therefore, fall-dormant alfalfa is sensitive to light signal variations, altering its growth pattern into a survival pattern, maintaining its dormancy pattern.

Evidence suggests that starch degradation may play a significant role in enhancing freezing tolerance during the early cold acclimation phase, during which degradation produces maltose, then produces glucose, which are then converted into soluble sugars. Soluble sugars serve as cryoprotectants and signaling molecules that are involved in plant adaptation to cold stress [21,22]. In Arabidopsis, the starch content is reduced in leaves within 24 h under cold temperature conditions [23]. Low temperatures induce the reprogramming of carbon metabolism and promote the conversion of newly fixed carbon into sucrose rather than starch [24]. We observed that the starch content first rapidly accumulated under short daylight conditions (phase 2) in the fall-dormant alfalfa, only decreasing in the roots when the temperature cooled down (phase 3), and finally accumulated in the roots again, slowly at first; large quantities of starch were consumed in the non-dormant alfalfa (Figure 3). Sucrose was first consumed in the fall-dormant alfalfa in phase 2, then accumulated in leaves during phase 3, and was finally mass-synthesized in the roots in phase 4; in contrast, sucrose first accumulated in the non-dormant alfalfa leaves and was consumed in the roots (Figure 4 and Figure 5). During the entire cold acclimation process, the fall-dormant alfalfa showed a dynamic transformation from sucrose to starch and then from starch to sucrose; the complete opposite process occurred in non-fall-dormant alfalfa.

Among the eight kinds of soluble sugars, raffinose appears to be the most critical signal for inducing cold acclimation in alfalfa, as it was the sugar detected in the leaves of the fall-dormant alfalfa under short daylight conditions. Raffinose is synthesized using sucrose and inositol as substrates [25]; in the present study, sucrose was consumed and raffinose accumulated in the fall-dormant alfalfa leaves during the short daylight phase (Figure 4 and Figure 5). However, no evidence of raffinose accumulation in the non-dormant alfalfa was observed; instead, inositol was synthesized in the non-dormant alfalfa leaves under the short daylight conditions, which might have generated an erroneous sugar signal. Li et al. [26] recently showed that *ZmRAFS* might mediate galactinol hydrolysis into inositol in leaves if there is sucrose insufficiency in plants. The sucrose content was indeed lower in the non-dormant alfalfa than in the fall-dormant alfalfa during the growing season, and it might have been insufficient for raffinose synthesis in the second phase, which is in line with this phenomenon. Our results suggest that the sucrose content during the growing season is a key factor influencing the development of cold acclimation in alfalfa, such that the production of raffinose can induce fall-dormant alfalfa to begin cold acclimation in a timely manner. This provided evidence for a central role of sucrose metabolism in carbon allocation during alfalfa cold acclimation [27].

**Figure 7:**
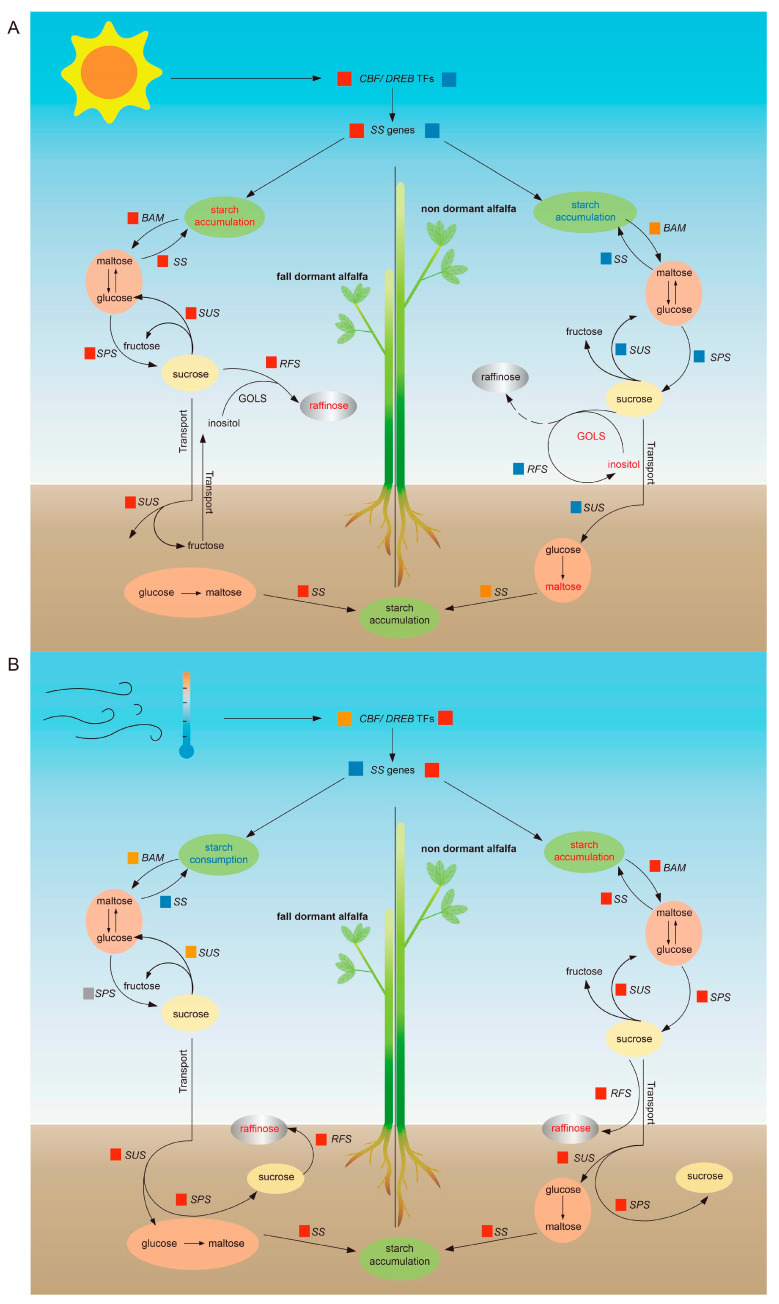
Saccharide transformation and regulation strategies of two alfalfa cultivars with different fall dormancy levels. Note: (**A**) shows saccharide transformation and regulation strategy during short daylight period, (**B**) shows saccharide transformation and regulation strategy during cooling phase; the squares represent gene expression, with red: upregulated, to yellow: non-significance, to blue: downregulated. The arrow points to the direction of transformation between different saccharides. The dotted line indicates failure of raffinose synthesis.

By integrating all the data, we clearly identified that saccharide transformation occurred in the fall dormancy alfalfa (Figure 7). Compared with the non-dormant alfalfa, *SUS*s were upregulated in the fall-dormant alfalfa during the short daylight phase, leading to sucrose hydrolysis into fructose and glucose, so that the sucrose content was reduced, and the fructose and glucose contents increased. Maltose was subsequently produced, the *SS* expression was upregulated, and the activity of starch synthase encoded by the *SS*s was enhanced; maltose was then converted into starch, causing the starch content to increase. The *SUS* expression was higher than that of *SS*, the biological process of sucrose hydrolysis predominated, and saccharide transformation occurred in the fall dormancy alfalfa, with sucrose converted into starch under sunlight signal changes. *RFS* was also simultaneously upregulated, and sucrose was used to produce raffinose, inducing alfalfa’s response to the cold acclimation process. *BAM*, *SUS*, and *SPS* were upregulated in the roots until the cooling period, and this resulted in starch’s hydrolysis into maltose and then into glucose. The glucose and fructose were then converted into sucrose, which accumulated in the fall dormancy of alfalfa. Notably, the entire process was dependent on the regulation of *CBF/DREB1A* TFs [28].

In brief, based on high sucrose accumulation in the growing season, alfalfa can sensitively respond to natural light changes. Sucrose was transformed into starch for energy reserves, and raffinose was synthesized for stress-resistance regulation. Subsequently, starch was then transformed quickly into sucrose during the cooling period, which is critical for cold acclimation and developing cold resistance. This study is a real field study, and the conclusion has relevant applicability; from its results, we can better understand the mechanism of alfalfa’s cold tolerance development. However, its limitation is that the varieties used in this study can only be used to compare the overwintering rate for the next year; thus, it is impossible to compare the agronomic traits and yields of the two varieties for the next year.

## 4. Materials and Methods

### 4.1. Plant Materials and Treatments

Two alfalfa cultivars, *Medicago sativa* L. ‘Sardi No. 10’ (SD, fall dormancy rating = 10) and *Medicago sativa* L. ‘Zhaodong’ (ZD, fall dormancy rating = 1~2), were studied. SD served as the control group. Seedlings were initially established in flowerpots (6 cm × 6 cm × 11 cm) at a greenhouse in an experimental station at the Institute of Grassland Research, China Academy of Agricultural Sciences, where the mean annual temperature is 5.8 °C and the mean annual precipitation is 390 mm. The soil type was defined as chestnut soil, and this region exhibits a temperate monsoon climate. Each cultivar was planted across 45 flowerpots. One seed was planted in each flowerpot, and seedlings with consistent growth were kept after 7 days of emergence. These seedlings were subsequently individually transplanted with a distance between plants of 50 cm × 50 cm; 5 plots of each cultivar were chosen randomly. Conventional field management techniques were used. The initial flowering stage of alfalfa in Hohhot was roughly the first ten days of July, at which point the cultivars were taken and mowed.

The observations and sample collections were started on the first day of August and ended on the last day of November, capturing the transition from growth to cold to freezing temperature stress conditions. The period included control phases in August (phase 1 served as the control group), September (phase 2, with short daylight conditions), October (phase 3, with cooling conditions), and November (phase 4, with temperature under 0 °C); the monthly average temperature and monthly average illumination time are shown in Figure S20. Sampling was performed on the 21st day of each month.

The whole plant was taken and divided into its aboveground part and belowground part, used for plant height and root diameter measurement and dry weight determination, according to the methods of Zhao et al. [29]. For dry weight determination, the aboveground and belowground samples were rinsed and dried naturally, then put into an oven and dried at 105 °C for 30 min, then dried at 65 °C until reaching a constant weight. Each phase was designed with three biological repeats, and each biological repeat was designed with three technical repeats, which were obtained by cutting propagation. The overwintering rate was evaluated in the subsequent year when growth resumed.

Leaf and root samples were collected for physiological analysis, transcriptome analysis, and reverse-transcription quantitative PCR. The sixth fully expanded leaf sample for alfalfa at each phase was collected from the top and bottom of the alfalfa plants, and axial root samples were collected in the same period; these samples were immediately put in liquid nitrogen once taken for transport to the laboratory and then stored at −80 °C until use.

### 4.2. Physiological Index Determination

To investigate soluble sugar accumulation in the two alfalfa cultivars during cold acclimation, the concentrations of starch and eight other soluble sugars—maltose, sucrose, fructose, glucose, sorbitol, inositol, trehalose, and raffinose—were determined. Samples were extracted from the leaf and root tissues. The starch content was determined as described by Smith et al. [30], in which eight soluble sugars were determined in accordance with the manufacturer’s protocol (Solarbio, BC4614, BC2465, BC2455, BC2505, BC2525, BC0330, BC5840) [31]. For brevity, a 0.1 g sample and 1 mL of distilled water were added and ground; next, centrifugation at 12,000 rpm for 10 min was performed and the supernatant was taken to be tested; and the absorbance was recorded at wavelengths of 480 nm, 520 nm, 505 nm, 655 nm, 600 nm, and 700 nm, respectively. The raffinose content was determined according to the ultra-performance liquid chromatography (UPLC) method described by Xu et al. [18]. Briefly, a 0.1 g sample was used, incubated according to the corresponding instructions, extracted using 0.5 mL extraction buffer, centrifuged for 10 min at room temperature, and the supernatant was used for determination. Raffinose, in a 0.1 g frozen sample, was determined and extracted by adding 0.7 mL of 80% ethyl alcohol solution and heating at 70 °C for 2 h. The liquid supernatant was purified with chloroform. Soluble sugars were separated and analyzed on an Ion Chromatography System 5000 instrument (Thermo Fisher Scientific, Waltham, MA, USA) equipped with a Hypercarb PA20 column (Thermo Fisher Scientific) with a mobile phase consisting of ddH_2_O and 200 mM NaOH at a flow rate of 0.5 mL·min^−1^. The raffinose concentration was quantified based on external standards.

### 4.3. Enzymatic Activity Assay

Sucrose synthase (SuSy), soluble starch synthase (SS), α-amylase (α-AMY), and galactinol synthase (GolS) activities were determined using the anthrone assay, as described previously [23,28]. Briefly, leaf and root materials were suspended in an extraction buffer containing 50 mM HEPES–KOH (pH 7.5), 10 mM MgCl_2_, 1 mM EDTA, 2.5 mM DTT, 10% (*v*/*v*) glycerin, and 0.1% (*v*/*v*) Triton X-100. After incubation on ice and centrifugation, the supernatant was used for the assay. First, the extraction of SS and α-AMY was performed at room temperature (22 °C), and the extraction of SuSy and GolS was performed at 4 °C, which has been reported to be the optimum temperature. The reactions were performed at 30–37 °C for 10–15 min by heating in a water bath, and stopped at 90 °C for 5 min, then cooled down to 4 °C. Absorbance of samples was measured at room temperature under 540 nm, 620 nm, 450 nm, and 405 nm, respectively.

### 4.4. Transcriptome Sequencing

Total RNA was extracted from the root samples using TRIzol reagent (Invitrogen, Waltham, MA, USA) following the manufacturer’s instructions. The purity, concentration, and integrity of the samples were assessed using Nanodrop Qubit 2.0 (both from Thermo Fischer Scientific, Waltham, MA, USA) and an Agilent 2100 Bioanalyzer (Agilent, Santa Clara, CA, USA) to guarantee the eligibility of the samples for sequencing. All samples were sent to Biomarker Co., Ltd. (Beijing, China), and six cDNA libraries (including three replicates) were constructed and sequenced using the HiSeq 2000 platform (Illumina, San Diego, CA, USA). Transcriptome assembly was accomplished using Trinity software v2.15.1, and gene function was annotated based on the NR, Pfam, KOG/COG/eggNOG (Clusters of Orthologous Groups of proteins), Swiss-Prot, KEGG, and GO databases. Gene expression was estimated by RNA-Seq using expectation maximization, and differentially expressed genes (DEGs) were analyzed using DESeq^2^ (https://bioconductor.org/packages/release/bioc/html/DESeq2.html, (accessed on 9 November 2020)).

### 4.5. Analysis of Gene Expression by Reverse-Transcription Quantitative PCR (qRT-PCR)

Total RNA was extracted using TRIzol reagent (Invitrogen, Waltham, MA, USA) following the manufacturer’s instructions. First-strand cDNA synthesis was performed using a FastKing gDNA Dispelling RT SuperMix Kit (Tiangen, Beijing, China) according to the manufacturer’s protocol. Specific primers for qRT-PCR were designed using Primer 5.0 software (PREMIER Biosoft, San Francisco, CA, USA) and synthesized by Sangon Biotech Co., Ltd. (Shanghai, China). qRT-PCR analysis, with three biological replicates per sample, was performed using a QuantStudio 6 real-time PCR system (Applied Biosystems, Waltham, MA, USA) with 2×SG Fast qPCR Master Mix (Low Rox) (Sangon Biotech, Shanghai, China). *AFD2* was used as an internal reference gene for normalization. The primers used for RT-qPCR analysis are listed in Table S23. Gene expression was calculated using the 2^−∆∆CT^ method.

### 4.6. Statistical Analyses

The data were subjected to an analysis of variance between treatments using IBM SPSS Statistics 20.0, and figures were generated using Origin 2021. Figures and statistical representations were assembled using Adobe Illustrator 2019.

## Figures and Tables

**Table 1:** Statistical results of differentially expressed gene (DEG) numbers.

Treatment	Upregulated	Downregulated	Total DEGs
ZD Leaf phase 1 vs. SD Leaf phase 1	1018	1628	2646
ZD Root phase 1 vs. SD Root phase 1	4980	6822	11,802
ZD Leaf phase 2 vs. SD Leaf phase 2	4199	3171	7370
ZD Root phase 2 vs. SD Root phase 2	4260	3408	7668
ZD Leaf phase 3 vs. SD Leaf phase 3	3629	1410	5039
ZD Root phase 3 vs. SD Root phase 3	5033	3285	8318
ZD Root phase 4 vs. SD Root phase 4	8731	7198	15,929

## Data Availability

The original contributions presented in this study are included in the article. Further inquiries can be directed to the corresponding author.

## Supplementary Materials

The following supporting information can be downloaded at https://www.mdpi.com/article/10.3390/plants14091313/s1, Figure S1: Comparative analysis of changes in the proportion of different soluble sugars; Figure S2: Comparison of gene expression levels under cold acclimation; Figure S3: Heat map of the Pearson correlation coefficient of each sample under cold acclimation; Figure S4: GO enrichment circle diagram of all leaves samples; Figure S5: GO enrichment circle diagram of all roots samples; Figure S6: SS DEGs in leaves of three phrases; Figure S7: SS DEGs in roots of four phrases; Figure S8: BAM DEGs in leaves of three phrases; Figure S9: BAM DEGs in roots of four phrases; Figure S10: SPS DEGs in leaves of three phrases; Figure S11: SPS DEGs in roots of three phrases; Figure S12: SUS DEGs in leaves of three phrases; Figure S13: SUS DEGs in roots of four phrases; Figure S14: RFS DEGs in leaves of three phrases; Figure S15: RFS DEGs in roots of four phrases; Figure S16: DEGs identification of DREB1A; Figure S17:DREB1A DEGs in leaves of three phrases; Figure S18: DREB1A DEGs in roots of three phrases; Figure S19: Validation of transcriptome sequencing data by qPCR analysis; Figure S20: Changes in duration of sunlight (h) and mean daily air temperature (℃) from August to November. Table S1:A summary of the transcriptome sequencing and assembly results in the fall dormant (ZD) and non-dormant (SD) alfalfa; Table S2: Go annotation of DEGs in leaves samples during phase 1; Table S3: Go annotation of DEGs in leaves samples during phase 2; Table S4: Go annotation of DEGs in leaves samples during phase 3; Table S5: Go annotation of DEGs in roots samples during phase 1; Table S6: Go annotation of DEGs in roots samples during phase 2; Table S7: Go annotation of DEGs in roots samples during phase 3; Table S8: Go annotation of DEGs in roots samples during phase 4; Table S9: KEGG annotation of DEGs in leaves samples during phase 1; Table S10: KEGG annotation of DEGs in leaves samples during phase 2; Table S11: KEGG annotation of DEGs in leaves samples during phase 3; Table S12: KEGG annotation of DEGs in roots samples during phase 1; Table S13: KEGG annotation of DEGs in roots samples during phase 2; Table S14: KEGG annotation of DEGs in roots samples during phase 3; Table S15: KEGG annotation of DEGs in roots samples during phase 4; Table S16: DEGs of DREB TFs in leaves samples during phase 1; Table S17: DEGs of DREB TFs in leaves samples during phase 2; Table S18: DEGs of DREB TFs in leaves samples during phase 3; Table S19: DEGs of DREB TFs in roots samples during phase 1; Table S20: DEGs of DREB TFs in roots samples during phase 2; Table S21: DEGs of DREB TFs in roots samples during phase 3; Table S22: DEGs of DREB TFs in roots samples during phase 4; Table S23: Primer sequence information for RT-qPCR.
