# Supplementary material for: Unveiling the Cold Acclimation of Alfalfa: Insights into Its Starch-Soluble Sugar Dynamic Transformation
Source: Plants (Basel). 2025 Apr 26;14(9):1313. doi: 10.3390/plants14091313 (PMC12073796; doi:10.3390/plants14091313)
Supplement: Supplementary file 1 [file plants-14-01313-s001.zip › plants-3577289-supplementary.pdf]

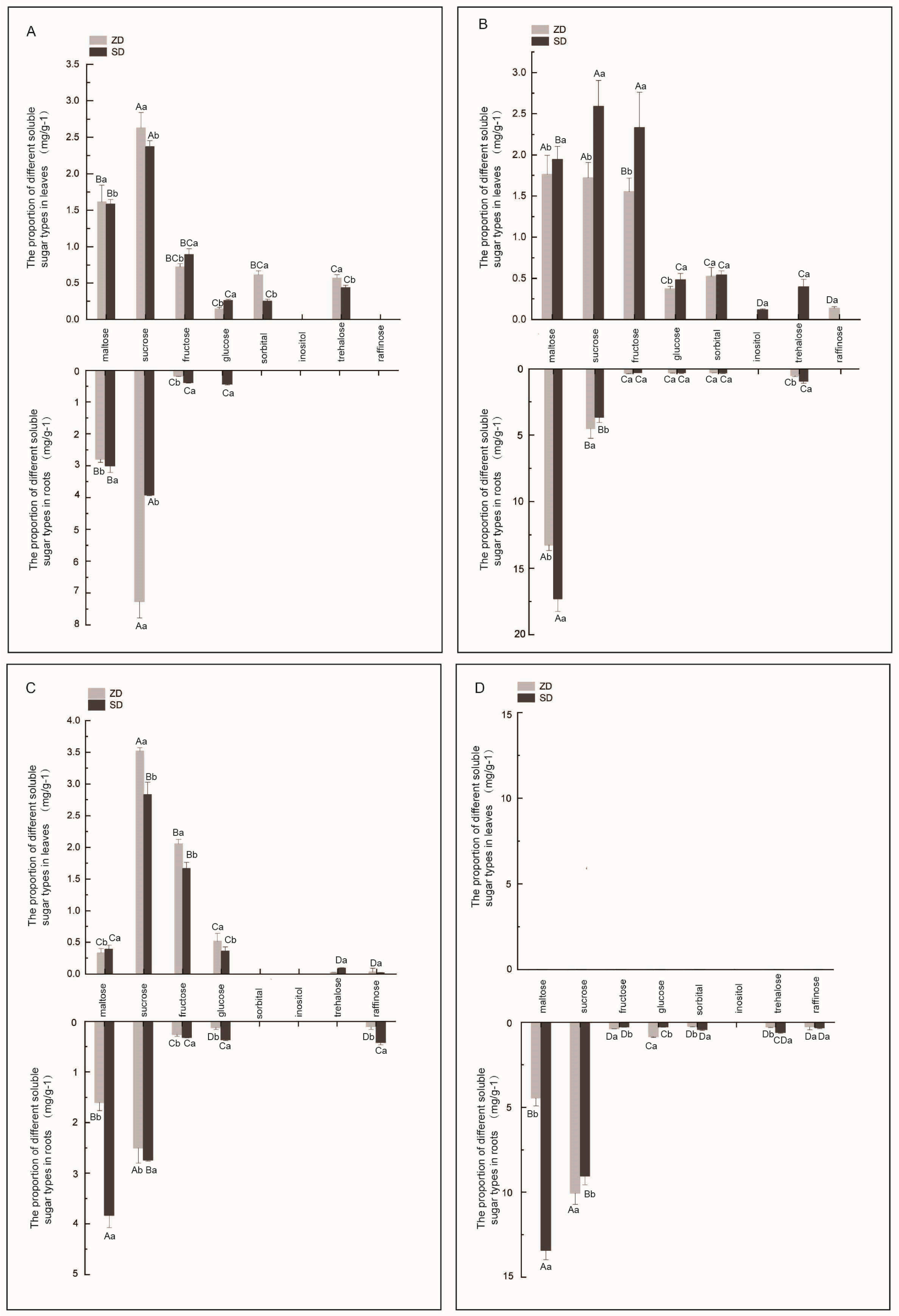

Figure. S1 Comparative analysis of changes in the proportion of different soluble sugars

Note: a, b, c and d represent the proportions of different soluble sugar types in the four phases of the two alfalfa cultivars, respectively.

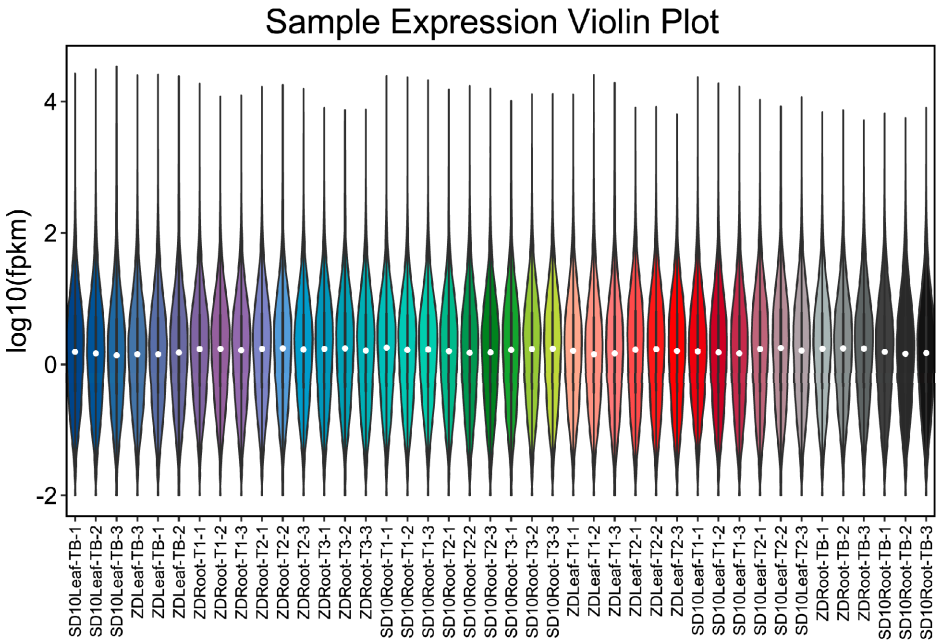

Figure. S2 Comparison of gene expression levels under cold acclimation

Violin plot showing the distribution of the FPKM values of each sample under low-temperature stress. The X-axis in the boxplot shows the ID of each sample. The Y-axis represents the log^{10} (FPKM).

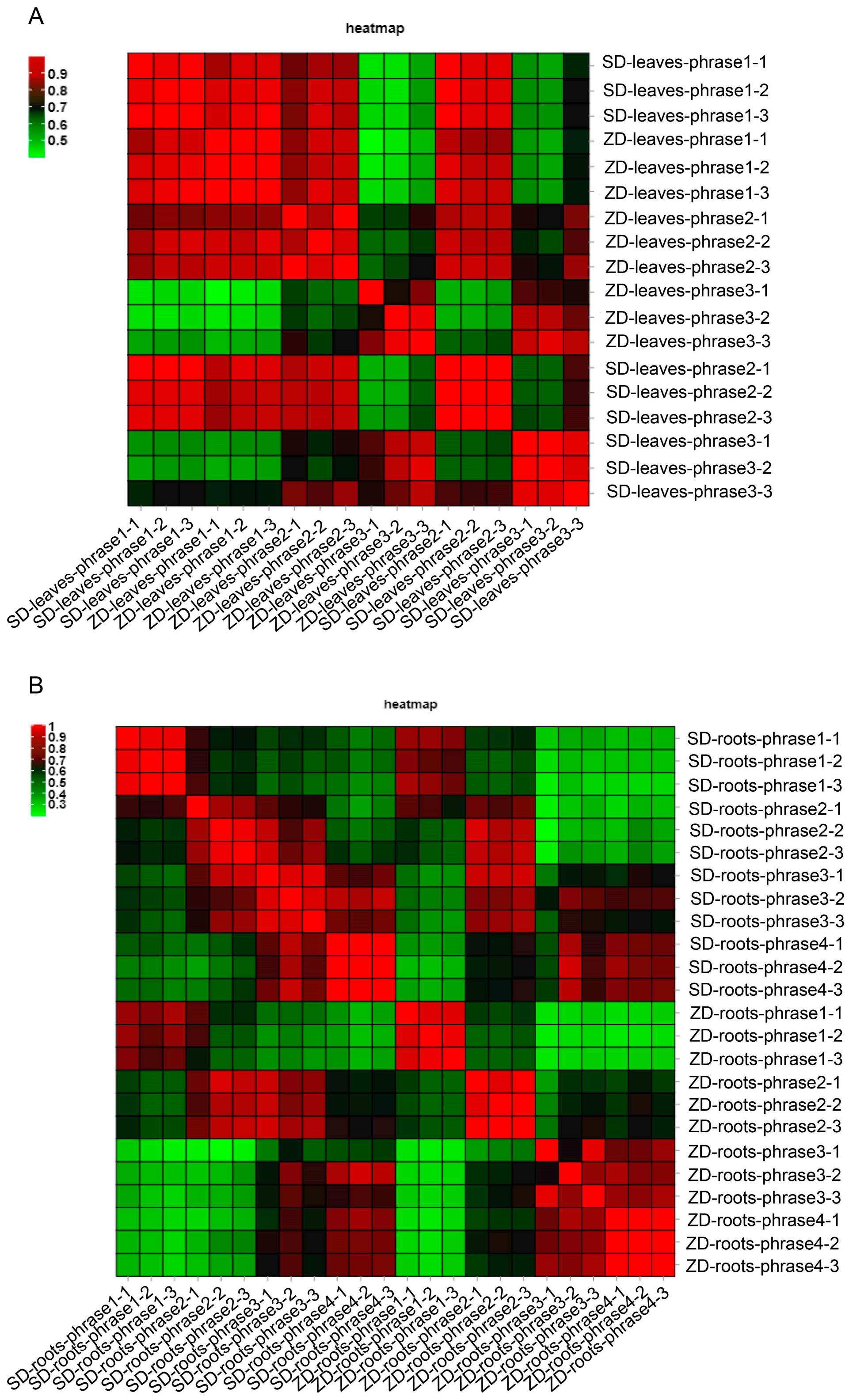

Figure. S3 Heat map of the pearson correlation coefficient of each sample under cold acclimation

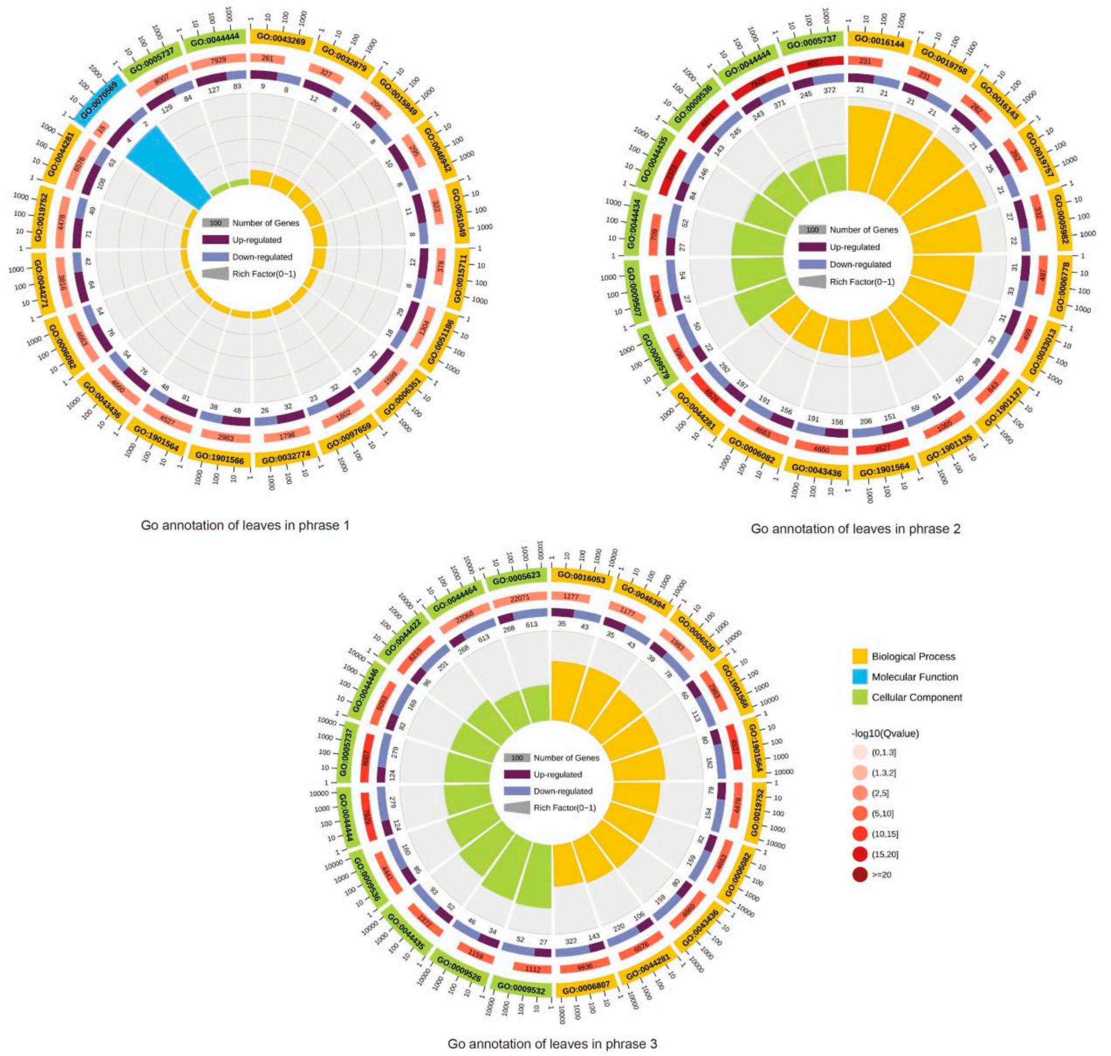

Figure. S4 GO enrichment circle diagram of all leaves samples

Note: The first circle: the first 20 GO terms of enrichment, the outside of the circle is the coordinate scale of the number of genes. Different colors represent different ontology. The second circle: the number and Q value of the GO term in the background gene. The more genes, the longer the strip, the smaller the Q value, the redder the color. The third circle: up-regulated and down-regulated gene ratio bar chart, dark purple represents up-regulated gene ratio, light purple represents down-regulated gene ratio ; the specific values are shown below. The fourth circle: rich factor value of each GO term (the number of differential genes in the GO term divided by all the numbers), background grid line, each grid represents 0.1).The same as below.

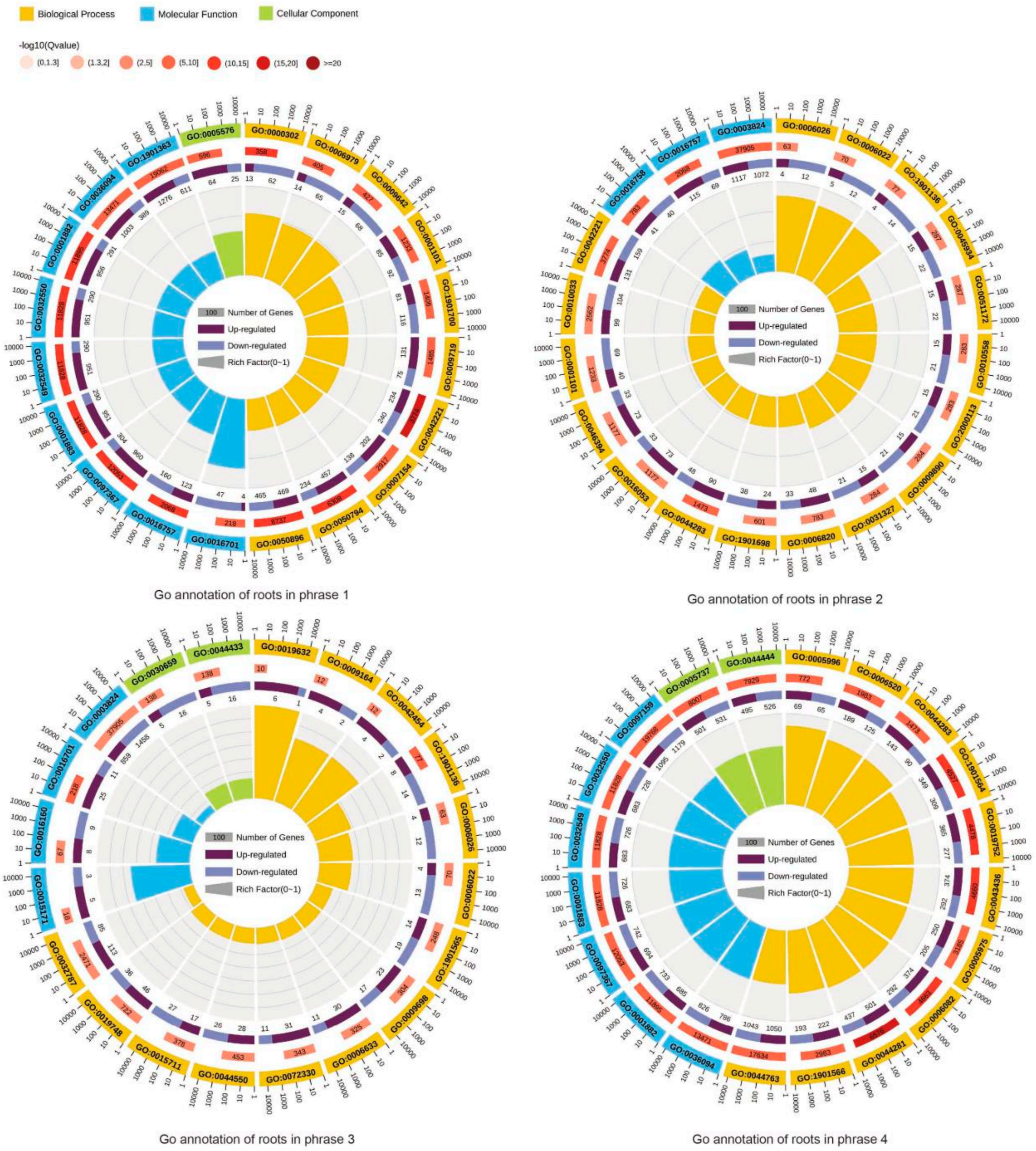

Figure. S5 GO enrichment circle diagram of all roots samples

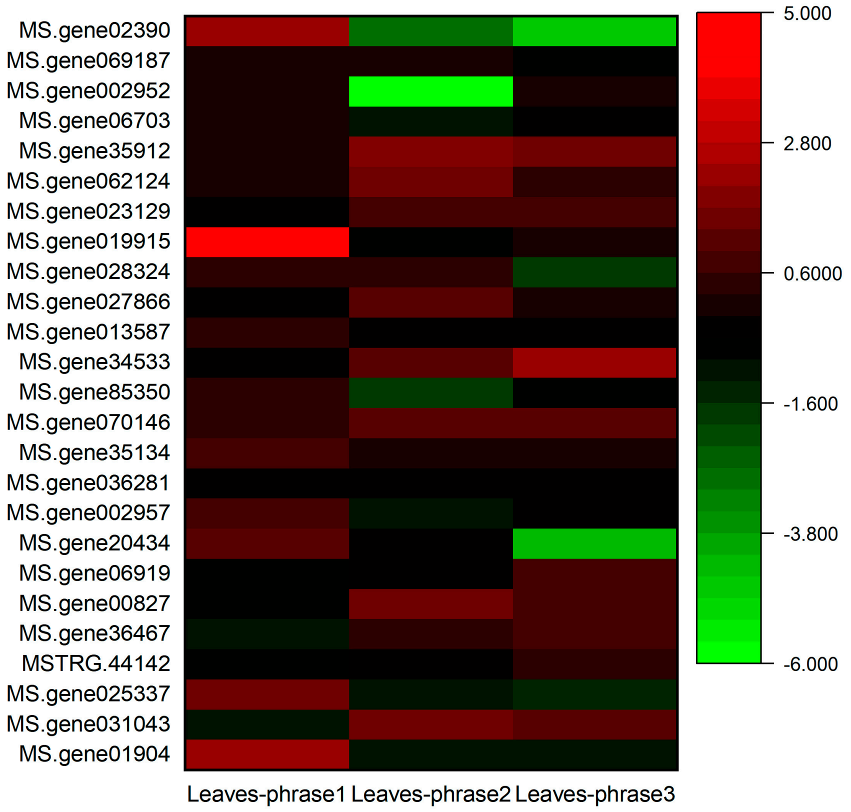

Figure. S6 *SS* DEGs in leaves of three phrases

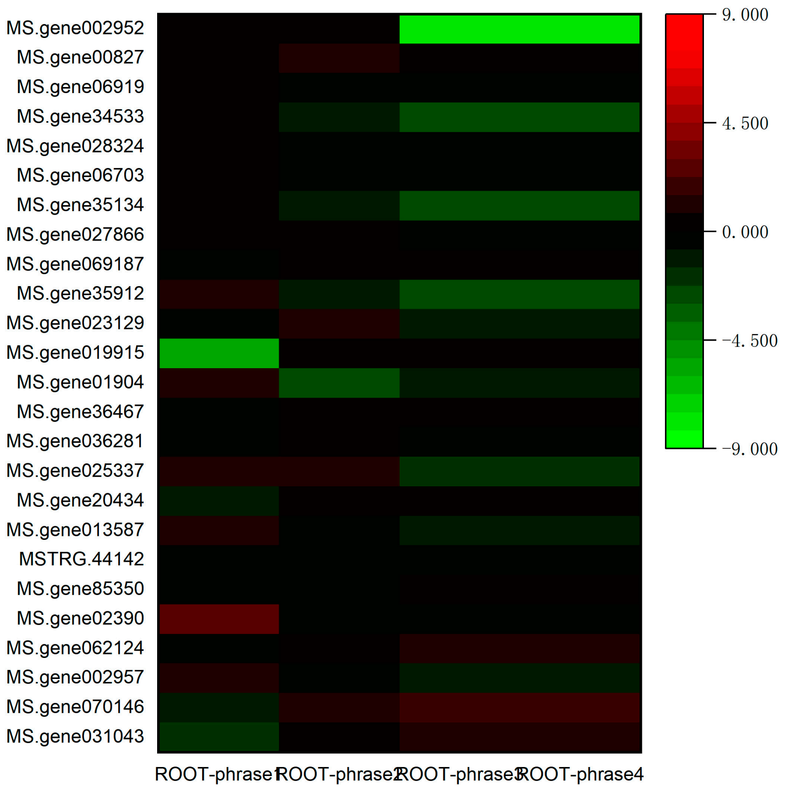

Figure. S7 *SS* DEGs in roots of four phrases

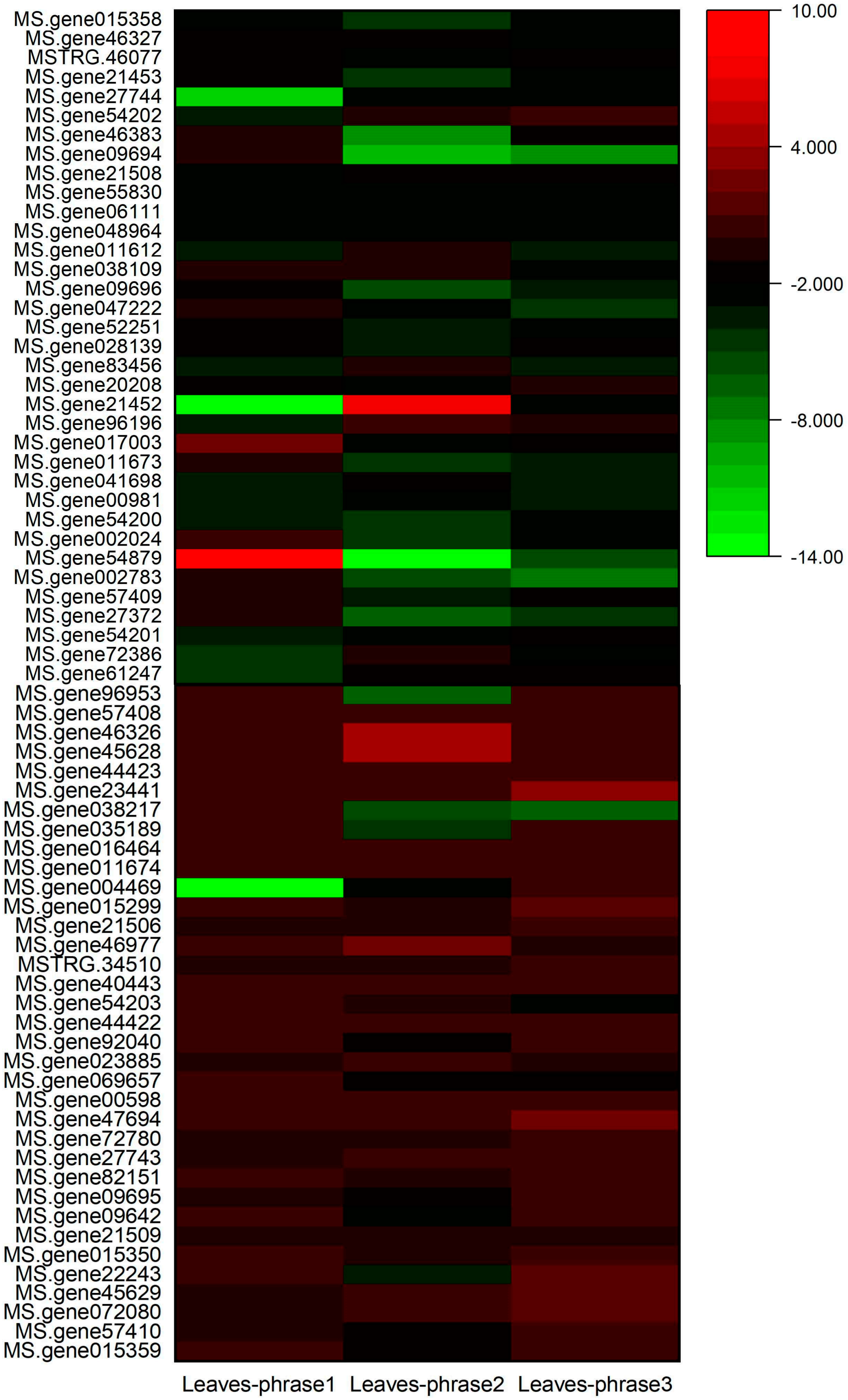

Figure. S8 *BAM* DEGs in leaves of three phrases

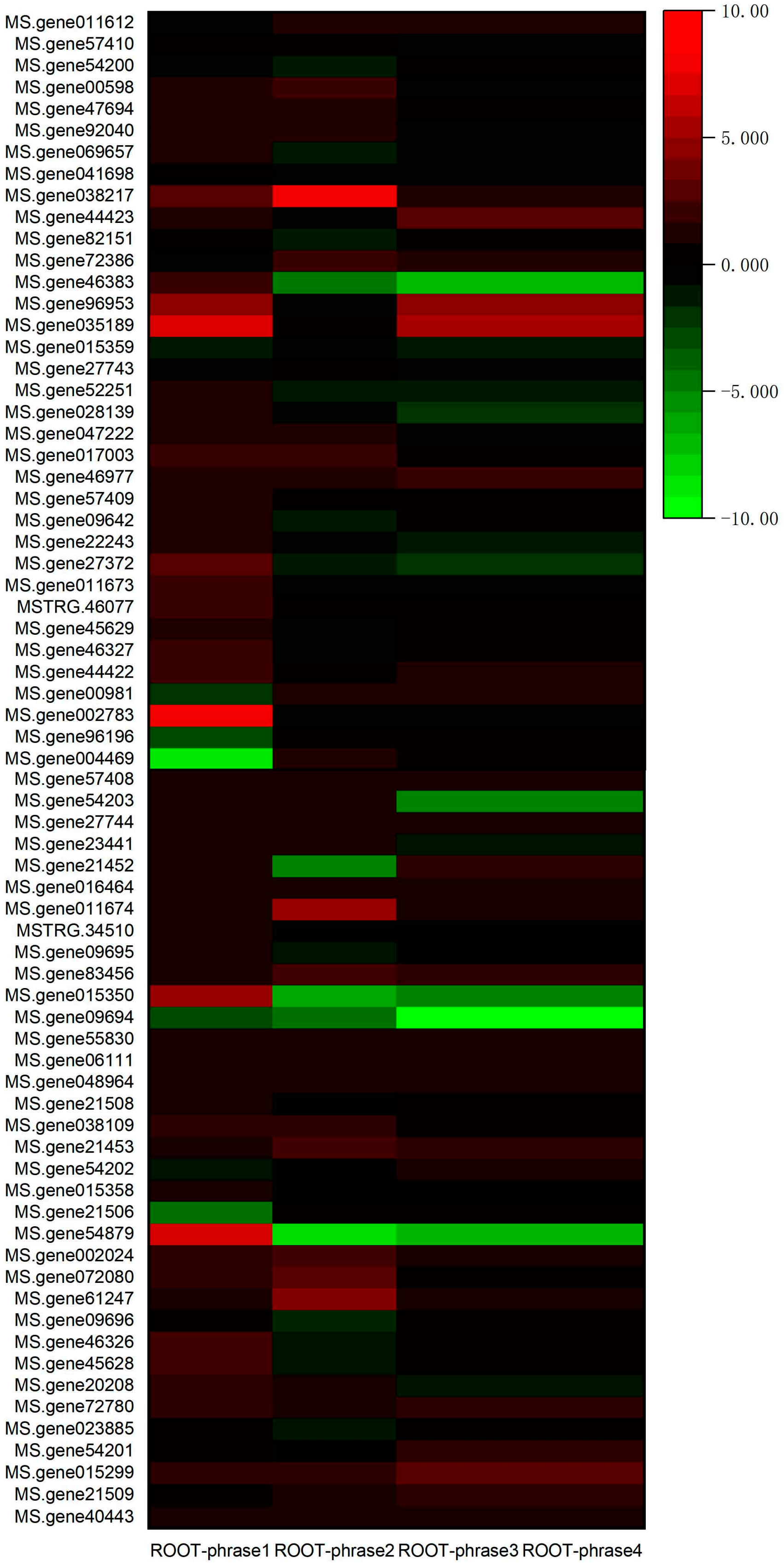

Figure. S9 *BAM* DEGs in roots of four phrases

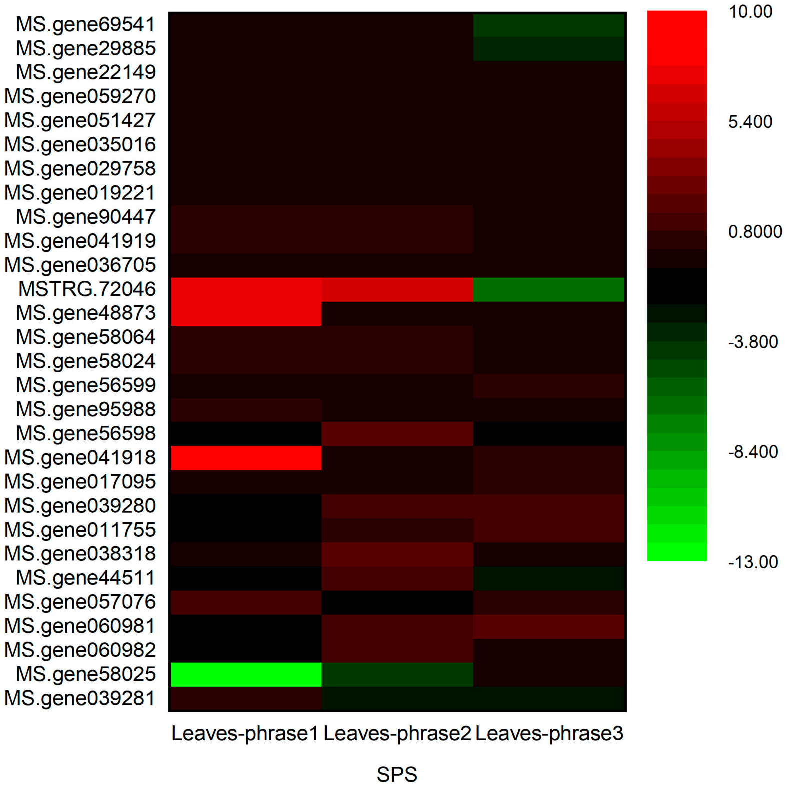

Figure. S10 *SPS* DEGs in leaves of three phrases

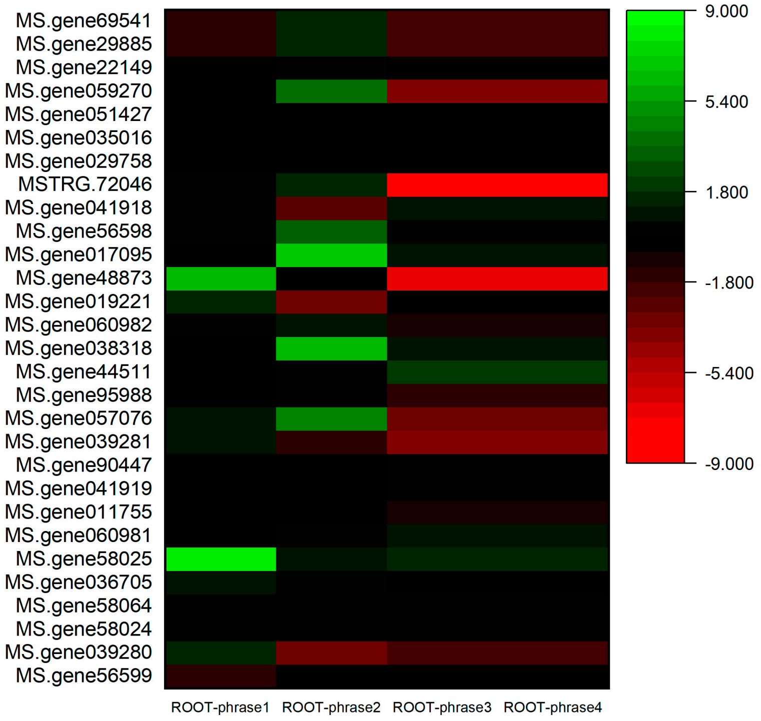

Figure. S11 *SPS* DEGs in roots of three phrases

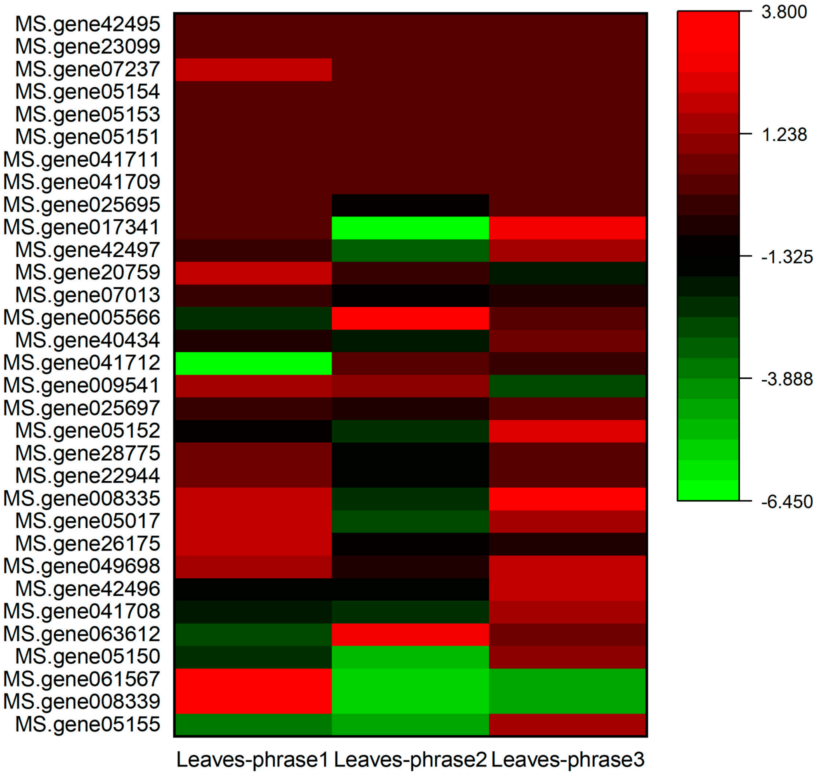

Figure. S12 *SUS* DEGs in leaves of three phrases

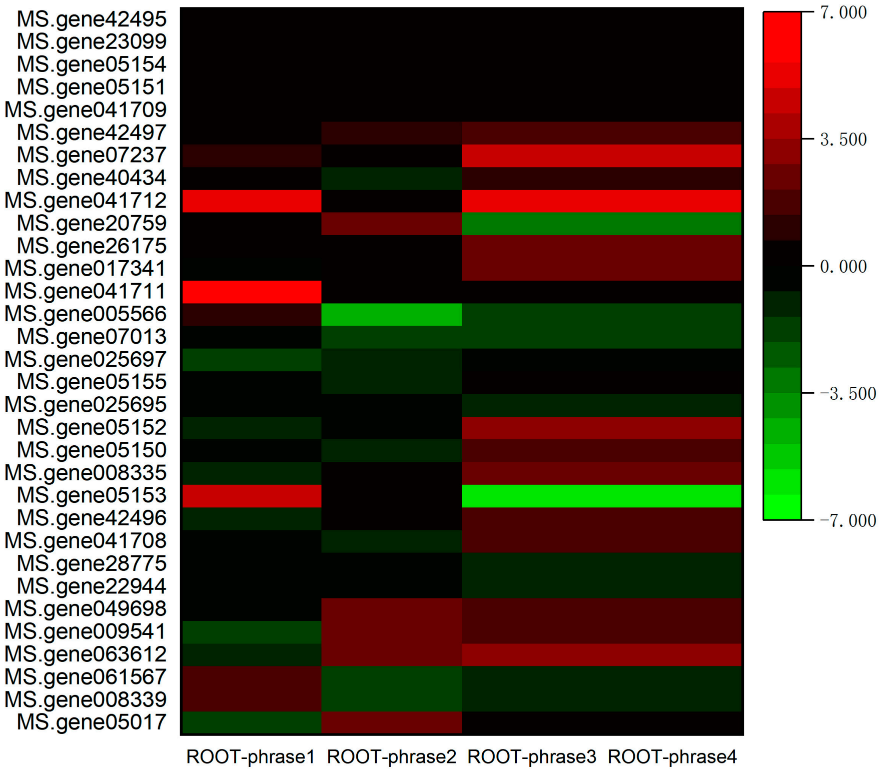

Figure. S13 *SUS* DEGs in roots of four phrases

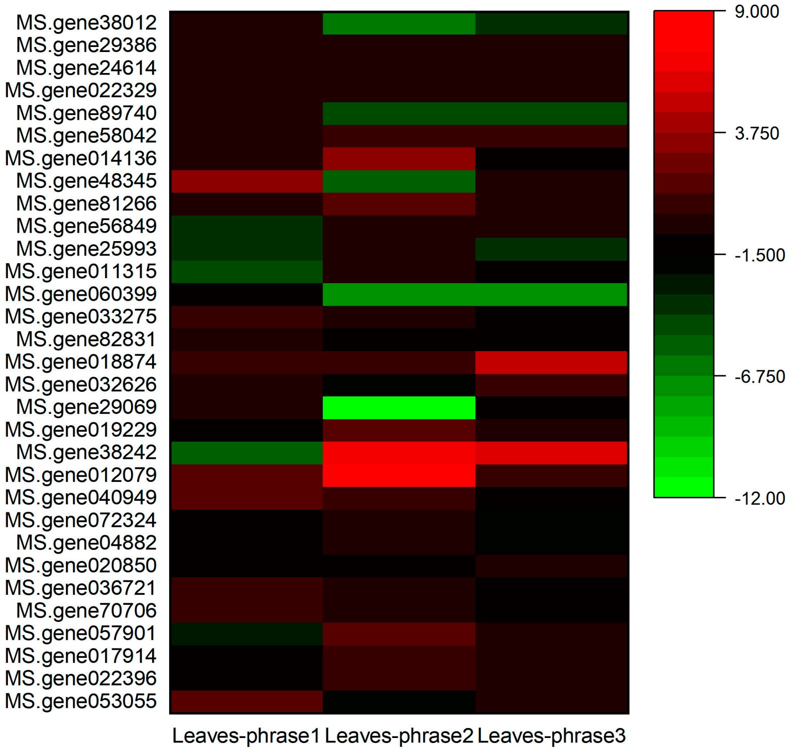

\

Figure. S14 *RFS* DEGs in leaves of three phrases

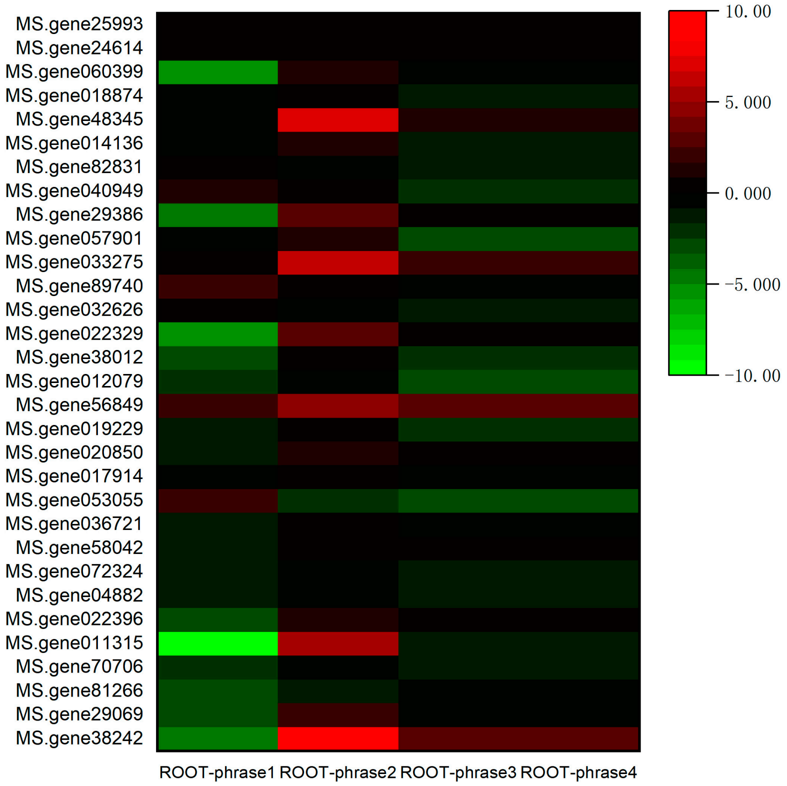

Figure. S15 *RFS* DEGs in roots of four phrases

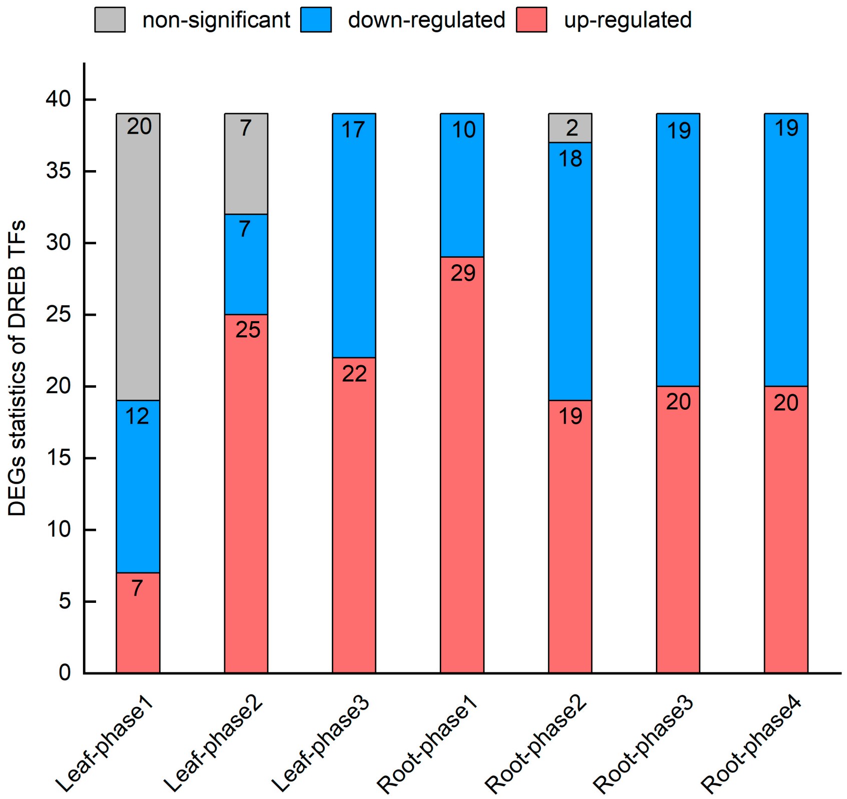

Figure. S16 DEGs identification of *DREB1A*

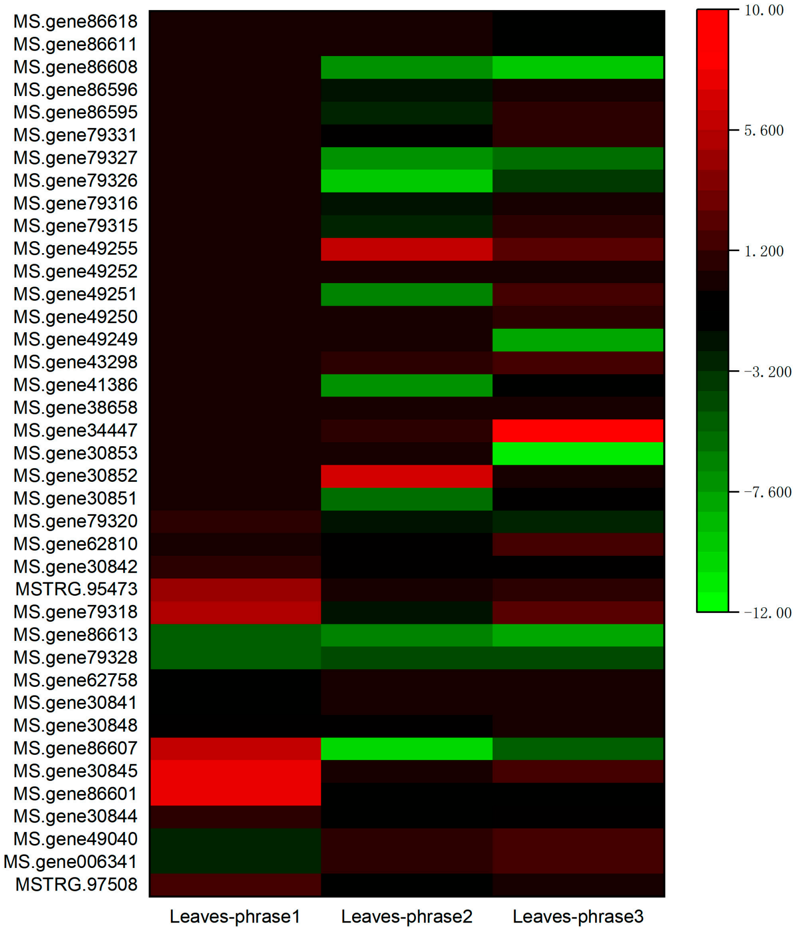

Figure. S17 *DREB1A* DEGs in leaves of three phrases

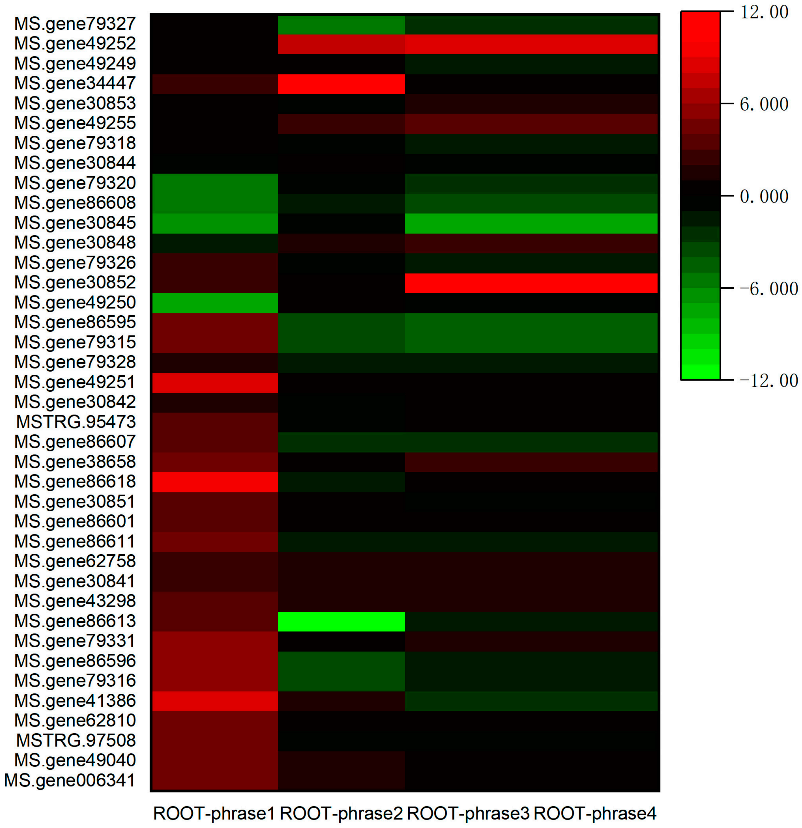

Figure. S18 *DREB1A* DEGs in roots of three phrases

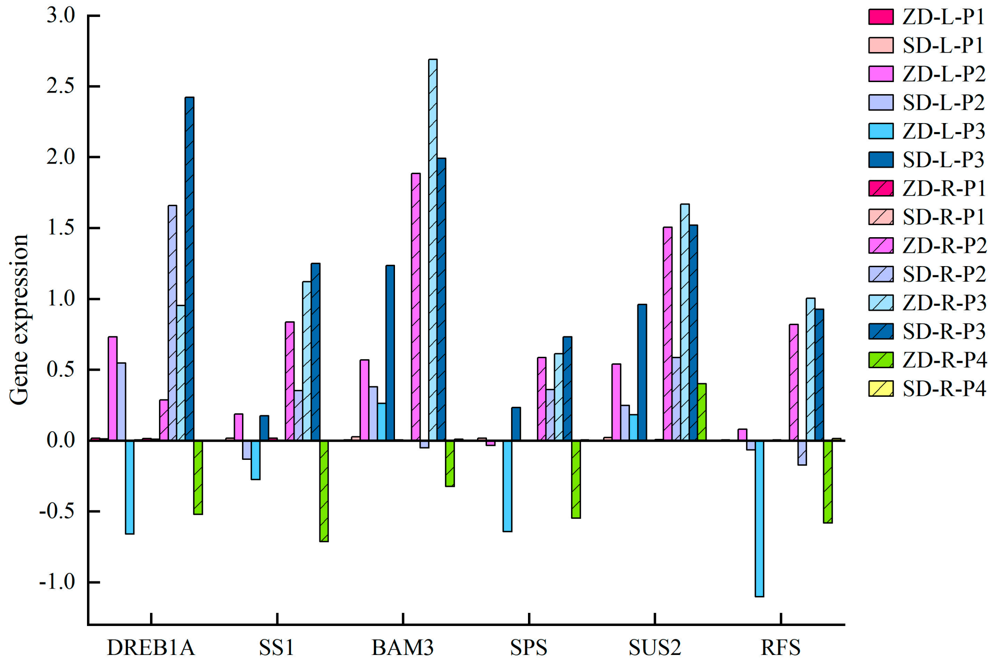

Figure. S19 Validation of transcriptome sequencing data by qPCR analysis

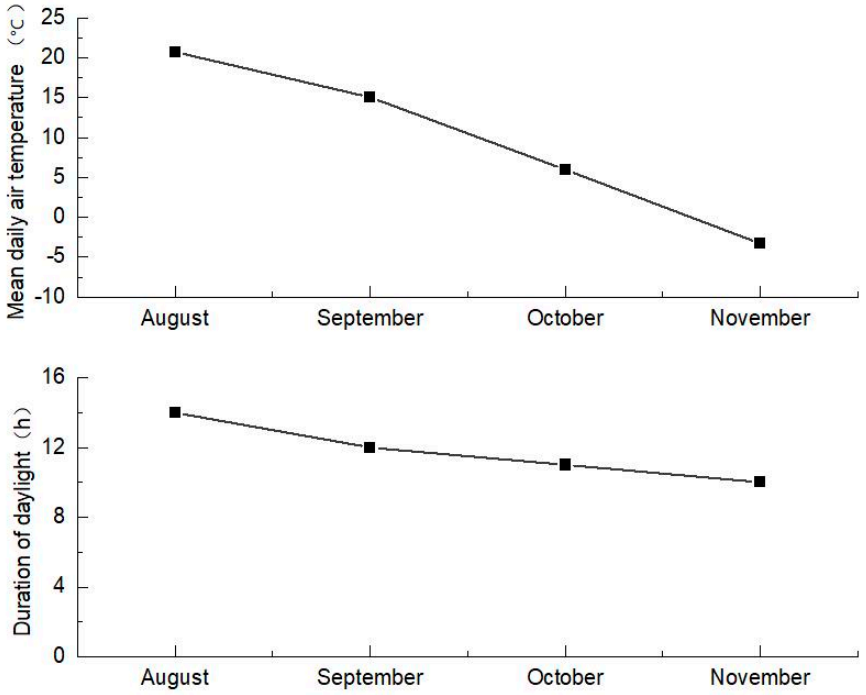

Figure. S20 Changes of duration of sunlight（h）and mean daily air temperature （℃）from August to November.
